# Health-Promoting Effects and Everyday Experiences With a Mental Health App Using Ecological Momentary Assessments and AI-Based Ecological Momentary Interventions Among Young People: Qualitative Interview and Focus Group Study

**DOI:** 10.2196/65106

**Published:** 2025-04-29

**Authors:** Selina Hiller, Christian Götzl, Christian Rauschenberg, Janik Fechtelpeter, Georgia Koppe, Eva Wierzba, Julia Sauter, Sina Dietrich, Daniel Durstewitz, Ulrich Reininghaus, Silvia Krumm

**Affiliations:** 1 Department of Psychiatry II, University of Ulm and BKH Guenzburg Guenzburg Germany; 2 Technical University of Munich, School of Medicine and Health, Department of Psychiatry and Psychotherapy, TUM University Hospital Munich Germany; 3 Department of Forensic Psychiatry and Psychotherapy, University of Ulm and BKH Guenzburg Ulm Germany; 4 Department of Psychosomatic Medicine and Psychotherapy, University of Ulm Ulm Germany; 5 Department of Public Mental Health, Central Institute of Mental Health, Medical Faculty Mannheim, Heidelberg University Mannheim Germany; 6 German Center for Mental Health (DZPG), partner site Mannheim-Heidelberg-Ulm Mannheim Germany; 7 Interdisciplinary Center for Scientific Computing, Faculty of Mathematics and Computer Science, Heidelberg University Heidelberg Germany; 8 Hector Institute for AI in Psychiatry, Central Institute of Mental Health, Medical Faculty Mannheim, Heidelberg University Heidelberg Germany; 9 Department for Psychiatry and Psychotherapy, Medical Faculty Mannheim, Heidelberg University Heidelberg Germany; 10 Department of Theoretical Neuroscience, Medical Faculty Mannheim, Heidelberg University Heidelberg Germany; 11 Faculty of Physics and Astronomy, Heidelberg University Heidelberg Germany; 12 Centre for Epidemiology and Public Health, Health Service and Population Research Department, Institute of Psychiatry, Psychology & Neuroscience, King’s College London London United Kingdom; 13 ESRC Centre for Society and Mental Health, King’s College London London United Kingdom; 14 Department of Psychiatry and Psychotherapy Leipzig University, Medical Faculty Leipzig Germany

**Keywords:** mobile health app, ecological momentary assessment, ecological momentary intervention, just-in-time adaptive intervention, digital training, mental health promotion, artificial intelligence, participatory research methods, user experiences, cocreation, young people, youth, adolescent, mHealth, interview, focus group, participatory approach, self-awareness, digital intervention, digital health

## Abstract

**Background:**

Considering the high prevalence of mental health conditions among young people and the technological advancements of artificial intelligence (AI)–based approaches in health services, mobile health (mHealth) apps for mental health are a promising way for low-threshold and large-scale mental health promotion, prevention, and intervention strategies, especially for young people. However, insufficient evidence on health-promoting effects and deficient user-centric designs emphasize the necessity for participatory methods in the interventions’ development processes.

**Objective:**

This study aimed to explore young people’s everyday experiences using an AI-based mHealth app for mental health promotion based on ecological momentary assessments and ecological momentary interventions. Our analysis of qualitative data focused on exploring young people’s use patterns in daily life and mental health–promoting effects.

**Methods:**

We conducted problem-centered interviews and focus groups with a subsample of 27 young people aged 14 to 25 years, who were among the participants of 2 microrandomized trials testing and evaluating an AI-based mHealth app (AI4U training). Our study used a participatory approach, with “co- and peer researchers” from the dialogue population actively engaged in research processes and data analysis. Structural content analysis guided the qualitative analysis.

**Results:**

Participants reported enhanced emotional self-awareness and regulation in daily life through the ecological momentary assessments and ecological momentary interventions. Young people appreciated the AI4U training for managing emotions and stress. They had no trust issues regarding disclosing their mental health via the AI4U training in daily life. Some faced challenges integrating it into their daily routines and highlighted the value of autonomy in use decision-making processes.

**Conclusions:**

Our findings reveal that young people benefited from enhanced emotional awareness and management through the use of the AI4U training, appreciating its anonymity for facilitating emotional disclosure. The results suggest that enhanced self-directed use may improve daily life integration, although participants noted that they sometimes avoided using the AI4U training during distress despite recognizing its potential benefits. These findings indicate the importance of balancing directed use and autonomy in digital interventions to harmonize compliance with effectiveness in daily life. We highlight the importance of participatory research for tailored digital mental health solutions.

## Introduction

### Digital Mental Health Interventions for Young People

According to the Global Burden of Disease study, almost 14 million young people in Europe aged 10 to 24 years in 2019 met the diagnostic criteria for a mental health condition [1]. The onset of most mental health conditions has been found to occur in youth or early adulthood, and there is an evident link between occurrence in early life and persistence or exacerbation in adulthood, highlighting the importance of early interventions [2-5]. Research calls for holistic and multi- and transdisciplinary approaches for mental health promotion (eg, improving resilience and strengthening protective factors) and more tailored interventions for young people [2]. There is evidence indicating that existing services are underused by young people due to social factors such as stigma, embarrassment, lack of trust in mental health professionals, and structural barriers related to the availability and accessibility of proper support [2,4,6-8]. The desire for self-reliance and autonomy grows from puberty into early adulthood and constitutes a further major barrier in seeking informal or professional help for mental health problems among young people [9].

Technological innovations provide new forms of efficient, large-scale, and low-threshold health services. With the growing accessibility to technology, smartphones have become powerful tools, making mobile approaches to mental health care and promotion services increasingly popular [10-13]. The widespread use of smartphones among young people makes them an attractive platform for mobile health (mHealth) apps or digital interventions aimed at promoting mental health through self-help features [14-23]. Evidence-based mHealth apps have the potential to target a wide range of users, promoting mental health and preventing mental health conditions in both clinical and nonclinical populations [10]. Most currently available reimbursable digital health applications in Germany meet the evaluation criteria for patient-relevant clinical benefits [24]. Digital self-monitoring of symptoms or behavior (eg, via ecological momentary assessments [EMA]) allows for the collection of ecological valid data on mental health outcomes and precise clinical assessment [11,21,25-27]. On the basis of EMA data, real-time and context-dependent personalized intervention suggestions (eg, ecological momentary interventions [EMI] or, synonymously, just-in-time adaptive interventions) for improving mental health can be provided to the user and subsequently evaluated [28-32]. Interventions that are based on real-time data may further increase user adherence and engagement and improve effects on mental health [10].

Research emphasizes the importance of personalization in mHealth apps as it simplifies the process for users to find suitable interventions and can improve the effectivity of the selected interventions [10]. Modern artificial intelligence (AI) methods pave the way for new and targeted forms of mHealth services [33-35]. AI is used for a variety of tasks, including risk prediction and prognosis, diagnostic classification, and personalization of interventions, among others [34,36]. In the context of personalizing mHealth apps, time-series models such as recurrent neural networks have been proposed to build a dynamic and predictive model of individual behavior based on which interventions can be optimally selected [32,35]. This allows for a personalized and adaptive allocation of EMI.

However, time expenditure and the potential of mHealth apps as an additional stressor in daily life have been recently identified as major barriers to daily mHealth app use among young people [37]. In addition, studies still face high attrition rates and low engagement from young participants, such as in the study by Peuters et al [15], in which young people reported neglecting several features of the mHealth app, mainly due to time constraints. Interviews with homeless young people using an EMA-based mHealth app revealed that repetitive and predictable questions and answer options quickly led to boredom among the participants [38].

Public health literature increasingly calls for research to guarantee active engagement with dialogue populations in the research process to enhance the acceptance and implementation of health promotion and prevention measures [39-44]. This approach ensures the inclusion and empowerment of affected individuals, including marginalized groups, in research processes that impact them and establishes transformative knowledge through the integration of new perspectives. Exploratory qualitative research and participatory approaches are essential to inquire about the existing gaps in understanding how mHealth app use can be optimized. Digital services, especially for young digital natives, require a collaboration on equal terms among and active participation by health care professionals, developers, and individuals from the dialogue population, and successful coproduction especially requires the integration of potential users early in the development process [11,17,45,46]. An in-depth understanding of expectations or experiences of people regarding digital mental health care approaches is crucial for the development of effective and trustworthy solutions [47].

However, qualitative studies on people’s real-world experiences with mHealth apps based on EMA have predominantly centered on specific groups, such as patients with bipolar disorder [48], patients with depression [49], or homeless young people [38], or on family research [50]. One study conducted interviews with college students on their experience with an EMA-based mHealth app aimed at improving emotional well-being [51]. However, the participation period was rather short, averaging 10 days, and the evaluation focused on methodological and design-related research questions rather than on individual effects on mental health. Qualitative data on the use of EMI tend to be lacking. One study investigated the effects of a mobile meditation intervention through participant interviews [52]. However, Xie et al [52] targeted adults diagnosed with clinically elevated levels of depression or anxiety. To our knowledge, no existing qualitative study using principles of participatory research has explored the subjective effects of an AI-based mHealth app on the mental health and emotional resilience in daily life of healthy young people.

### Study Objectives

The objective of this qualitative study was to explore the perspectives of young people (aged 14-25 years) on the use of an AI-based digital training administered via a smartphone-based app for personalized mental health promotion in youth based on EMA and EMI (AI4U training). We were particularly interested in their views regarding use and integration of the mHealth app into everyday life and the subjective effects on their mental health, especially health-promoting effects. The following research questions were addressed:

1. What are young people’s subjectively perceived health-related effects of the AI-based mHealth app?
2. How do young people perceive the use of an AI-based mHealth app designed to promote mental health in everyday life based on EMA and EMI?

## Methods

### Study Design

#### Study Background

This qualitative study formed part of the “AI4U—artificial intelligence for personalized digital mental health promotion in youth” living laboratory. Living laboratories strive to foster collaboration between science and society and engage target or dialogue populations and stakeholders from a diverse range of relevant sectors to explore and study innovative models of action such as innovations in health services [53]. The living laboratory AI4U was dedicated to the participatory development, optimization, and evaluation of an AI-based mHealth app for personalized mental health promotion in youth (AI4U training). The living laboratory involved the direct participation of relevant stakeholders, users from the dialogue population, and an interdisciplinary research group embedded in a transdisciplinary infrastructure to ensure ongoing quality management, sustainability appraisal, and dissemination. Optimization and initial evaluation of the AI4U training involved 3 separate consecutive microrandomized trials (MRTs) in line with the multiphase optimization strategy [54]. In the MRTs, participants aged 14 to 25 years were recruited and asked to complete the 40-day AI4U training using an mHealth app, with a target sample size of 60 participants for each MRT. In the 3 within-subject MRTs, participants were repeatedly randomized using a 1:1 ratio to either AI-based allocation of EMI components based on EMA ratings or to a random allocation of EMI components at a maximum of 210 decision points per participant to examine the effect of the AI algorithm on proximal outcomes in youth. EMA ratings consisted of questions on momentary (positive and negative) affect, activities (eg, physical activity), and perceived contexts (Multimedia Appendix 1). Further details on the living laboratory AI4U can be found in previous papers [55-57].

This study focused on qualitative data collected as part of the first 2 MRTs (MRT 1 and MRT 2) to allow for adaptation and optimization of the mHealth app based on subjective experiences. Due to the participatory approach of the AI4U living laboratory, young people from the study’s dialogue population were involved early in the study. A female researcher (SH) was employed as a “peer researcher.” This ensured the active participation of young people in many aspects of the decision-making processes (including study design, implementation, and dissemination). “Co-researchers” (JS, SD, and 2 others), that is, young people of the same age as the dialogue population with no pertinent qualitative research experience, were included in project-related research processes by taking part in “participatory research workshops”. Similar to research workshops held with qualitative researchers, these workshops aimed to increase the quality of data analysis through joint interaction [58,59]. Co-researchers met on a regular basis with the research team (SH and CG) to participate in qualitative research. To enable adequate participation, co-researchers received training on core competencies in qualitative research (eg, interviewing and data analysis) and were involved in various stages of the research processes throughout the study. Key elements were the participatory development of interview topic guides, joint moderation of focus groups (FGs), and subsequent discussion of preliminary results [56]. Similar participatory health research has shown that participatory evaluation processes with nonscientific co-researchers from a project’s dialogue population promotes the generation of transformative knowledge and facilitates shared decision-making in qualitative research, particularly between academic researchers and co-researchers [60].

#### Interventions

The AI4U training is a digital training that consists of four innovative forms of digital mental health promotion, which users can apply directly in their everyday lives: (1) EMA [25,26], (2) EMI [29,31,61], (3) AI [62-64], and (4) a web-based dashboard for digital monitoring and feedback. The AI4U training was delivered over a 40-day intervention period in addition to public mental health care as usual available through the German health and social care system. The digital training consisted of at least one face-to-face session with a coach and an EMI administered through a smartphone-based app for adaptive real-time and real-world transfer. The sessions with the coach provided guidance on the content and structure of the training and were complemented by optional sessions with counselors for young people receiving care from educational counseling services. The AI4U training consisted of a 10-day introductory period with self-monitoring using EMA (8 per day) and a 30-day training period with self-monitoring using 6 EMA per day. EMA entries could be viewed on a dedicated digital dashboard using a web browser. The AI4U training intended to improve resilience, fostering a compassionate self-image and strengthening self-care skills in everyday life (Multimedia Appendix 1). Following each MRT, the digital training was optimized further based on feedback from participants at the end of the study and data from qualitative interviews.

#### Sample

We recruited participants for the qualitative process evaluation consecutively among the young people who had used the AI4U training for the previous 40 days. For this, we used a purposive sampling strategy to select participants with relevant insights into the use and impact of the AI4U training. This method allowed us to specifically target individuals who met predefined criteria, such as various age groups or users with various degrees of adherence. In addition, we aimed for a balanced gender distribution and inclusion of young people from varying socioeconomic and educational backgrounds (Multimedia Appendix 2). We contacted participants via email to inform them of the purpose of our interviews and the possibility of study participation.

### Data Collection

We used the COREQ (Consolidated Criteria for Reporting Qualitative Research) checklist [65] to ensure a clear and thorough description of the qualitative research methodology (Multimedia Appendix 3). Problem-centered interviews (PCIs) were held between October 2022 and November 2023. The FGs were held between December 2022 and July 2023. All interviews were conducted remotely using a video-based online tool. Experienced researchers (SH and CG) conducted the PCIs. In line with our participatory approach, one of our co-researchers (JS and SD) moderated the FGs. They signed a confidentiality agreement a priori and were provided with a guide for remote FGs (Multimedia Appendix 4). To ensure the quality of data collection, we practiced the moderation beforehand in a participatory research workshop, and co-researchers were supported by one of our research assistants with adequate scientific expertise (SH and CG).

For the PCIs and FGs, we used semistructured topic guides (Multimedia Appendix 5). The topic guide for the FGs was developed together with our co-researchers during our participatory research workshops. Both interview methods covered questions on (1) the use of the AI4U training in everyday life, (2) the evaluation of the EMA and EMI, and (3) trust factors in the AI4U training. Although the interview topic guides largely overlapped, the PCIs focused on the subjective health-promoting effects of the AI4U training, whereas these aspects were not a primary discussion point in the FGs. We used Microsoft PowerPoint slides (Microsoft Corp) to visualize the EMI and the dashboard interface to facilitate feedback on the key content of the AI4U training. After each FG, there was a short discussion between the co-researcher and the co-moderator to reflect on first impressions or discuss potential field notes taken.

### Data Analysis

The PCIs and FGs were held in German, audio recorded, and transcribed verbatim. For the transcription, we used f4 automatic speech recognition (Dr. Dresing & Pehl GmbH). Transcripts were subsequently pseudonymized and revised by student assistants. SH conducted data analysis using structural qualitative content analysis [66,67]. After reading of and familiarization with the data material, particularly interesting or important text passages were highlighted. In accordance with the semistructured interview topic guides, we created thematic categories and corresponding descriptions of coding rules for each of the categories. We then started with open coding of data material, facilitating the exploration of previously undisclosed themes. This process was followed by a subsequent categorization of coded text passages according to the thematic categories previously defined. If emerging codes were not suitable to any of the previously defined categories, we inductively added new thematic categories or codes. We coded transcripts using the MAXQDA 2020 software (VERBI GmbH) [68]. Validation procedures within the data analysis process encompassed multiple stages. After reviewing the initial coding tree within our research team (SH, CG, and SK), we modified and structured it accordingly. The coding trees and passages from the FG transcripts were subsequently thoroughly examined and validated with the co-researchers (JS, SD, and 2 others) in 2 distinct participatory research workshops. We marked the codes and subcodes that emerged through cocreation and validation in the participatory research workshop in the coding trees of the MAXQDA files accordingly (Multimedia Appendix 6). In a third participatory research workshop, we examined all the text passages previously highlighted as interesting and discussed and interpreted their potential meaning together.

### Ethical Considerations

This study was funded by the Ministry of Science, Research, and Arts of the State of Baden-Württemberg, Germany (study period from January 2021 to June 2024), and approved by the Medical Ethics Review Committee II of Heidelberg University (Mannheim Medical Faculty; reference 2022-550). All participants provided written informed consent. For underage participants, we also obtained informed consent from parents or legal guardians. Participation in the study was voluntary. Consent could be withdrawn at any time without giving reasons. Participants were covered by liability and commuting accident insurance for the duration of the study. Participant data were analyzed and exchanged exclusively using study pseudonyms. The pseudonymized data were securely stored and processed in accordance with the General Data Protection Regulation. Participants received a compensation of €20 (US $22.40) in the form of a voucher redeemable at various stores across Germany.

## Results

### Sample Characteristics

Overall, 114 young people completed the 40-day AI4U training during the first 2 MRTs (N=57 in MRT 1 and N=57 in MRT 2). Of those 114 young people, we included 27 (23.7%; 13/57, 23% from MRT 1 and 14/57, 25% from MRT 2) in our qualitative study distributed across 16 PCIs and 3 FGs with 3 to 5 participants each (Multimedia Appendix 2). In the PCIs, the youngest participant was aged 14 years, and the oldest was aged 25 years (mean age 20.38, SD 3.78 years), whereas in the FGs, the youngest participant was aged 18 years and the oldest was aged 25 years (mean age 21.36, SD 1.84 years). Most participants had obtained their high school diploma or were already pursuing higher academic education (20/27, 74%). The average duration of the PCIs was 44 minutes, and the average duration of the FGs was 1 hour and 11 minutes. In the PCIs, gender distribution was even (8/16, 50% male individuals and 8/16, 50% female individuals). For MRT 1, we conducted 2 FGs divided by gender and with an equal number of participants (3 participants in each FG). The third FG with participants of MRT 2 was composed of 5 female individuals only.

### Overview

The following sections report on the subjective perspectives of participants on the effects of the AI4U training on their mental health, safety and trust considerations, the integration and role of the AI4U training in everyday life, and the value of self-directed use behavior. As the AI4U training was primarily administered through a smartphone-based app, participants used the terms *AI4U training* and *mHealth app* interchangeably in the interviews. Content within parentheses in the following sections reflects the relative frequency, and content within square brackets reflects attribution of participant statements made during the PCIs or FGs. *P* stands for *participant*. Multimedia Appendix 2 provides the participant characteristics.

### Subjectively Experienced Effects Following the AI4U Training

#### Mood Monitoring via EMA Seemed to Increase the Mental Health Awareness of Young People

Most participants (PCIs: 14/16; FGs: 7/11) reported that the awareness of their acute mental health status via EMA led to improved self-consciousness of emotions and subsequent self-reflection. One person especially appreciated the following:

...that it made you very aware of your own emotions. And just that, I found, often helped in some way, because then I frequently...had to think about how I was feeling at the moment. And I really liked that. Um, yes, just to become more aware of yourself and your own feelings.

PCI_P27; page 103

Participants mentioned that EMA were especially helpful to understand the reasons or contextual factors behind their emotions, that is, why they were feeling distressed (PCIs: 6/16; participant FG2_P1):

How do I feel, why do I feel this way? It makes you a bit more reflective. Understanding the reasons behind your emotions, why you behave like that, contributes to feeling better. I do feel that I learned something about myself through this.

PCI_P16; page 134

One interviewee argued that understanding contextual triggers enabled her to classify emotions on a more “objective level” (PCI_P16; page 60), whereas another participant (PCI_P13) highlighted that EMA helped reconsider recent emotional reactions as less severe upon further reflection.

Several young people (PCIs: 7/16; participant FG3_P3) highlighted that they aimed to sustain their mental health awareness over the long term, reflected more consistently on their daily experiences, and focused on the positive aspects in their everyday life even after the study period:

But I have to say, after I returned the [study phone], I had this feeling of emptiness [laughs]. Simply because I somehow mentally got used to these [EMA]. So, whenever something happened during the day, I immediately thought, “Okay, this has contributed to me being like a four out of seven in terms of my disappointment or something.” So, it definitely became ingrained in my mind.

FG3_P3; page 55

Some participants (PCIs: 6/16; FGs: 3/11) reported being interested in their long-term behavior or emotional trajectory and accessed the dashboard rather at the end of the study period. They described the dashboard and its visualizations of EMA responses as interesting but with rather low value relevant to their mental health promotion (PCIs: 6/16; FGs: 3/11):

[The dashboard]...was and is not a surprise because these are things that I have learned about myself for a long time. That's why I simply believe that it wasn't so surprising to see it, because I've repeatedly thought about these things before.

PCI_P26; page 182

However, some participants (PCIs: 5/16; FGs: 4/11) reported that they would have likely used the dashboard more frequently if it had been integrated into the app and not only accessible via a browser on a laptop.

#### EMI Appeared to Improve Emotion Regulation and Health-Promoting Routines in the Daily Life of Young People

According to a number of participants (PCIs: 7/16), EMI assisted in regulating emotions more effectively:

...I just felt mentally calmer and maybe you didn’t see the problem as the central problem, but everything was put into perspective a bit and it, um, yes, it mentally brought me down a bit and calmed me down.

PCI_P22; page 186

Through improved emotion regulation, several participants (PCIs: 3/16) mentioned learning that emotions, especially negative ones, were transient. For 2 of the 27 of the participants (PCI_P12 and PCI_P22), this health-related knowledge was retained in the long term. One participant reported that the EMI “Emotions as a Wave” (Multimedia Appendix 1) was particularly helpful:

I’ve always seen emotions as something that keeps coming back and stays, not something that fades away. But “Emotions as a Wave” is something that has changed my perspective on emotions itself because...it taught me that emotions can also pass and that resulted in me becoming a calmer person, actually.

PCI_P12; page 100

The breathing intervention was used as a health-promoting routine for relaxation and composure throughout the day by several individuals (PCIs: 5/16; participants FG3_P3 and FG3_P4) even after completing the AI4U training. Many participants (PCIs: 10/16; FGs: 6/11) reported that the “Moments of Joy” and “Positive Data Log” interventions stimulated positive affect by helping them focus on the enjoyable aspects of a day and diverting their attention from the negative ones. Another participant described the “Moments of Joy” intervention as helpful in becoming more “self-efficacious” (PCI_P14; page 188), realizing the extent to which she could also influence her own happiness or satisfaction. Another 2 of the 27 participants (PCI_P14 and PCI_P28) reported that they became aware of the importance of breaks in their daily lives:

...Whenever I have school assignments, I let them influence me a lot. I knew that before, but I’ve learned a bit that they shouldn’t completely fill my daily life, that I should still give myself more time. And the app helped me in setting that up somehow.

PCI_P28; page 212

When comparing EMA with EMI, a few participants (PCI_P17, PCI_P18, and FG2_P2) emphasized the particular benefit from EMA, whereas one described the EMI as “nice to have” but not necessary (FG2_P2; page 122). However, others (PCIs: 3/16) argued that perception of emotions via EMA was their first step toward change. Some participants (PCIs: 3/16) noted that EMA alone would be insufficient to develop new health-promoting routines. One participant reasoned that awareness of their own emotions enabled them to better manage them through the proposed EMI:

[The EMA have] caused me to simply become more aware of how I feel. And I think that led to better understanding these feelings and, as a result, either changing or accepting them. So, yes, just being able to handle them better...And especially the [EMI] could often make a difference there. For me, it was really often with nervousness that the [EMI] helped, for example.

PCI_P27; page 211

### Young People’s Trust Factors on Data Disclosure During Use of an mHealth App

#### Transparent Data Handling Seems to Be a Crucial Factor for Young People’s Confidence in Data Protection

As long as young people were confident that their data would not be misused, they reported that data disclosure via smartphones was not a use barrier (PCIs: 3/16; FGs: 3/11). A total of 4 of the participants (PCI_P15, PCI_P28, FG1_P2, and FG2_P2) stressed that transparency about the use of their data was crucial. Another 2 participants (FG1_P2 and FG1_P1) found it acceptable for the app to collect data only based on voluntary input and expressed concern about the collection of information that was not self-entered. Some participants (PCIs: 4/16; participant FG2_P3) felt more secure using the AI4U training because it was developed by professionals and used within an official study. In comparison, mHealth apps from an app store were deemed as less trustworthy by 2 participants (PCI_P28 and FG2_P3).

Some argued that, given the vast amount of personal information on the internet, data disclosure is something one “can’t prevent anyway” (PCI_P22 [page 233] and PCI_P21 [page 48]), and for others, it “doesn’t matter now anyway” (PCI_P21 [page 48] and FG2_P1 [page 97]). In total, 2 participants (PCI_P17 and PCI_P26) emphasized that one is used to providing personal information on their smartphone in everyday life. Therefore, it did not feel as if one was conveying information to someone else:

I didn’t really feel like I was sharing anything, but rather that I was doing it for myself...I mean, I’m used to handling a smartphone and I always write my notes on the iPad. So, it wasn’t strange for me to type something on a device.

PCI_P26; page 63

In total, 2 participants reported not perceiving the AI4U training as “invasive,” particularly when compared to algorithms on social media (FG3_P3 [pages 259 and 263] and FG3_P4 [page 287]). Some (PCIs: 3/16) noted that the app could probably not make any meaningful use of the personal data anyway. One interviewee emphasized her autonomy and personal agency in data sharing:

I’m generally not the type of person to think, “Oh God, data privacy,” and that something private will be stolen from me, because I still feel like I’m an independent person. If an AI knows more about my emotions, I don’t know how it could influence me in the long run. I just don’t see the risk there.

PCI_P26; page 67

#### Anonymity as a Necessary Requirement for Sharing Emotions

One participant noted that one had to “get used to” to being asked about their mental health or described it as an unfamiliar “change” (PCI_P15; page 4). For some (PCIs: 4/16; participant FG1_P3), disclosing emotions via the EMA felt unusual at first:

I first had to get used to the idea that there’s now an app, to which I should somehow describe my feelings, in some tasks that was the case. That was definitely something to get used to, but...somehow it subsided a bit, I would say. You just really got used to the fact that there’s no human there. Yeah, but it was a bit strange at the beginning...

FG1_P3; pages 94-116

Many participants (PCIs: 9/16) argued that they developed trust in the AI4U training because they did not perceive it as a real person or a human stranger. According to several participants (PCIs: 4/16), this significantly facilitated the expression of emotions:

So [it] definitely made it easier [to trust the app]. I believe I would have had a lot more reservations, if I had known that it was a human responding to me, especially because it would be a person I don’t know. And an AI is still an AI, even if your subconscious mind sometimes forgets that, but I think it definitely made it easier for me to talk about it and to overcome that barrier.

PCI_P27; pages 249-251

Young people reported feeling that their data were more securely handled than with humans and highlighted the barriers to sharing emotions during face-to-face communication as a reason (PCIs: 6/16; participants FG1_P3 and FG2_P1). Reported concerns of some young people (PCIs: 3/16; participant FG2_TN1) focused, for instance, on the fear of nonacceptance, judgment, and intimate disclosure typical in interactions with humans. According to some of them, the AI4U training was “just” an app that, unlike friends, could not misuse the data about their mental health:

On one hand, I felt safe, so to speak, because I know that an AI can’t really do much with [my information]. If I tell a person about it, I’m exposing myself to the person, and I don’t know what the person does with the information. And that wasn’t the case with the AI.

PCI_P16; page 36

Certain participants (PCIs: 6/16) reported that an mHealth app allows for a certain emotional distance and anonymity as a prerequisite for being open about their own emotions:

...you don’t see the direct reaction of people. The app accepts that. You can input everything and I believe you can be more honest with such an app, than if a person would ask me when I'm really feeling bad in a situation, “How are you?” I would probably still say, “Yeah, I'm fine,” but with the app, well, nobody really gets to know...It provides a bit more anonymity, I would say.

PCI_P14; page 42

The AI4U training was sometimes described as a “neutral diary” and catch basin for emotions that provided feedback based on objective data, not subjective assessment, for instance, compared to feedback from a therapist (PCIs: 4/16). Another participant (PCI_P12) identified this neutral feedback as stabilizing and more reliable and accurate, in contrast to human feedback.

### Integration of AI4U Training Into Young People’s Everyday Lives

#### Role of the mHealth App in the Daily Lives of Young People

Given that some participants (PCIs: 6/16) reported having a strict daily routine and everyday stress, they mentioned that they expected the AI4U training to assist them by helping reduce daily stress and monitor their mood. Some participants (PCIs: 6/16; FG3_P4) emphasized that they would not have paused their daily routine without the prompt from the AI4U training:

The app allowed me, so to speak, to take breaks, because it prescribed them to me. So, I think that when such an impulse comes from outside, telling you “now do it,” you’re more likely to do it, otherwise you always find an excuse...

PCI_P14; page 164

One participant highlighted that this heightened the awareness of her daily life structure and the importance of self-care within it:

In daily life, nothing reminds me...to check on my well-being. It doesn’t mean that my everyday life is very bad, but it’s about the fact that my everyday life is sometimes so hectic that I forget to take care of my well-being, my mental state...We’re so focused on our tasks that we forget about ourselves...and the app reminded one...to pay attention of what one should actually take care of, because the basic mindset shapes your everyday life. If you’re not doing well, you won’t perform at school, for example.

PCI_P12; pages 34-50

Several young people (PCIs: 6/16; participants FG3_P3 and FG3_P2) pointed out that they preferred using the AI4U training when experiencing discomfort in daily life. One participant remarked that she completed the EMA more frequently when experiencing negative emotions:

I feel like I tended to do them more when I wasn’t feeling well...And when I was feeling good, I thought, “Oh well, I don’t need to enter it now, I’m doing fine.”

PCI_P23; page 102

In total, 2 participants (PCI_P27 and FG3_P3) considered it less necessary when the app suggested interventions when they were feeling well. Another 2 participants (PCI_P17 and PCI_P22) reported that, when they were in a positive mood, they clicked through the EMA more quickly and superficially. One participant reflected on the AI4U training as rather an option to be accessed on an ad hoc basis in times of acute need:

I do think it could be a permanent companion, since one doesn’t have to use it every day, if you realize, today I don’t need it, today I’m fine.

PCI_P14; page 172

Without an urgent need, young people noted that they prioritized social interactions, especially if their daily life rhythm was quite strict, for example, due to work or educational duties (PCIs: 11/16; participant FG3_P1):

...I prefer to go to yoga or something similar and think about [my emotions] there, rather than having to fill out on my phone six times a day how I feel, where it just fell short. Eventually, I already knew the questions by heart and just clicked through them quickly.

FG3_P1; page 295

One participant (PCI_P29) mentioned refusing the AI4U training in acute situations of stress or negative feelings, preferring suggestions when he calmed down moments later. During stressful times, 2 participants (PCI_P23 and PCI_P15) were skeptical about repeatedly entering their emotions into EMA or EMI. However, another participant (FG2_P2) reflected that he neglected the app’s suggestions in challenging situations even when they could have been helpful.

Some participants (PCIs: 7/16) noted that the health-promoting routines they had learned during the AI4U training were practiced less frequently after the study period. Others (PCIs: 3/16) observed that, without a reminder, it was more difficult to maintain enough self-discipline for engaging in health-promoting routines. According to 2 participants (PCI_P14 and PCI_P23), habits were difficult to change, especially with increased stress and demands in everyday life. A few participants reported being less inclined to disrupt their routines (PCIs: 3/16):

I just think my daily routine is quite pre-structured with things that I can’t change fundamentally. So, I have to go to school when I have to go to school...I wouldn’t say that [the AI4U training] has restructured anything in that regard.

PCI_P28; page 204

In total, 2 participants (PCI_P24 and PCI_P25) felt that they might not be in as great need of mental health training compared to young people with actual mental health conditions:

It felt more for, uhm, sick people...who have serious problems in everyday life...I believe that it is much better received by them than it is by us (mhm), because it sometimes just felt like it wasn’t tailored exactly to us. So, what I expected was not what it turned out to be.

PCI_P24; page 27

#### Experiences With and Challenges Integrating the AI4U Training Into Daily Life

Overall feedback on daily life integration of the AI4U training was mixed. Most participants argued that app use was feasible when being at home or alone or when having a flexible daily rhythm (PCIs: 11/16; participants FG1_TN2 and FG2_TN2), and more challenging when they were on the go, in public, at work, or at school (PCIs: 10/16; FGs: 8/11). According to some participants (PCIs: 7/16), EMA or EMI were skipped when they spent time with their friends or family. A few participants (PCIs: 3/16; FGs: 4/11) reported feeling uncomfortable when they had to justify smartphone use to friends who perceived it as impolite, or when the alarm disturbed others. A total of 2 participants (PCI_P15 and PCI_P28) expressed discomfort or concerns about others witnessing them and judging their engagement with the intervention:

I somewhat refrained from EMI, especially at school or in public, as I was concerned about what my classmates might think if suddenly I close my eyes and sat at my desk.

PCI_P15; page 18

Some participants (PCIs: 5/16; FG2_P2) indicated that use significantly depended on an individual’s daily rhythm and was linked to the time of day and adaptability of daily duties. Several participants (PCIs: 8/16; FG2_P1) noted that a self-determined daily routine made it easier to integrate the AI4U training. Some (PCIs: 6/16; FGs: 3/11) reported that, when time was limited or the environment was unsuitable, EMA or EMI were either rushed through or skipped completely. A few participants (PCIs: 3/16) observed that, as the AI4U training became part of their daily routine, its use became less demanding.

Some participants (PCIs: 6/16; FGs: 3/11) reported EMA as less time-consuming and easier to fit into their daily lives compared to EMI. However, they reported that it was dependent on the perceived complexity of the EMI. For instance, a few (PCIs: 3/16; FGs: 3/11) perceived the “Breathing Intervention,” “Moments of Joy,” and “Positive Data Log” interventions (Multimedia Appendix 1) of the AI4U training appealing due to their simplicity, the lack of mental strain, and the feasibility to integrate them into daily life. More complex interventions such as “Emotional Compass” were often considered too challenging to apply in a daily life context, resulting in limited impact (PCIs: 9/16; FGs: 5/11). Others argued that EMI required increased mental effort and, consequently, more time (PCIs: 3/16; FGs: 5/11):

I declined EMI more often than EMA, because I found them more challenging to integrate into everyday life, especially while being on the go.

PCI_P27; page 179

Some participants agreed that the alarm of the EMA prompts was sometimes distracting (PCIs: 6/16; FGs: 6/11) and 6 EMA per day were too demanding to be conducted regularly as requested (PCIs: 3/16; FGs: 5/11):

I probably wouldn’t have needed [the EMA] for myself so frequently. Maybe it would have been enough for me two times a day, in the morning or evening, or perhaps midday, and then an EMI in the evening. That would probably have been the best approach for me.

PCI_P22; page 178

One participant (PCI_P28) mentioned that she felt bad declining prompts, whereas others reported that they felt “pressured” (PCI_P23; page 318) or “confined” by the app (FG2_P2; page 118). As a consequence, a number of participants (PCIs: 6/16; FG3_P4) described that they sometimes felt forced to conduct the AI4U training. A few (PCIs: 4/16; FGs: 4/11) sometimes perceived the AI4U training as an additional stressor:

It was difficult to integrate the app into my daily life in a way that made sense, that I can take away what they wanted to provide me, like doing EMI and such. For me, it was more of a stress factor (laughs) that I should take a break and do the EMI...

FG3_P1; page 43

As reported by some (PCIs: 8/16), it was burdensome to pause or interrupt ongoing activities in their daily lives. One participant noted having felt distracted by the alarms:

I also found it a bit distracting...When you’re trying to focus, you’re repeatedly pulled out of that focus to do [the EMA or EMI]. I found it a bit challenging to sit down in the library and start doing breathing exercises. So, I have to say, integrating it into my daily routine was relatively difficult and, I must admit, a bit tiring.

PCI_P24; page 19

### Young People’s Preferences Regarding Flexible and Self-Directed AI4U Training Use

#### Young People Demand Autonomy in Daily AI4U Training Use Behavior

From the perspective of some individuals, the temporal postponement of the EMA and EMI (PCIs: 5/16; participants FG1_P3 and FG2_P3) or muting the alarms (participants PCI_P27, FG1_P2, and FG1_P3) should be more flexible. Several young people (PCIs: 7/16; FGs: 7/11) expressed a desire for more self-determination in use behavior, such as having a stored calendar with personalized availability and preferred use times. Others (FGs: 5/11) suggested options for self-selection, for instance, via 2 variants of an EMI or a shortened version that they could choose from.

A few participants (PCIs: 9/16; FG1_P1 and FG3_P4) argued that increased self-determination could improve daily life integration and reduce the pressure, which some individuals experienced during the study period:

I believe it would be better for me personally to use it, if I had more control over inputting information myself...But given the frequency with which the app is now, with the EMA almost five times a day, I just don’t think that’s feasible for me.

PCI_P23; page 302

One participant (PCI_P23) remarked that the AI4U training had to fit into her daily routine without altering her habits or making her feel compelled to do so. Another participant emphasized that denying a prompt was always an option, and therefore, she had less of a problem with the app’s suggestions:

I mean, we always had the option to do the exercises ourselves...You always have the option to simply decline. Then you can still choose your own exercise.

FG3_P3; page 249

#### Young People Seem to Value Control in Content Selection and Decision-Making Processes

A few participants (PCIs: 3/16; FG3_P3) mentioned that they did not want to rely entirely on the app’s decision-making processes and expressed some doubts regarding the app’s ability to consistently capture their emotions accurately:

I think it’s quite individual. I also don’t believe that, to be honest, it is possible in the long run for [the app] to adapt to everyone, to all [personality] types. I can’t quite imagine that right now.

PCI_P25; page 166

Consequently, certain participants demanded an active role in influencing the degree of personalization, for instance, through a feedback function on the individual perceived usefulness of the EMI (participants PCI_P15 and PCI_P17; FGs: 3/11). In total, 3 participants from the FGs highlighted the importance of being able to deviate from the app’s content suggestions (FGs: 3/11). One participant emphasized the value of the interplay between self-directed use and app suggestions:

I would find it quite good if there was perhaps an app that still allows for some personal control despite AI. Because of course, the AI is hugely advantageous as it suggests something and maybe recognizes patterns that one might not have noticed, and providing EMI accordingly, but perhaps an additional option would be good, where you could say, “No, I don’t feel like doing this EMI, I would prefer to choose another one.” Sometimes you don’t really know which exercise is appropriate, and then the AI might have an advantage because it has already collected data.

FG1_P1; page 124

Some of our participants (PCIs: 4/16; FGs: 3/11) underscored that meaningful change can only occur through individual initiative. They emphasized a certain degree of willingness to change and openness to self-reflection as prerequisites for long-lasting change in health-promoting routines:

I believe that an app can indeed accomplish something like that. However, as a human being, you must have the ability. You have to be able to receive information or something and work with it...The app can definitely [help], if you look at the dashboard and are a reflective person with a lot of self-awareness who can work very well with oneself, then definitely.

PCI_P12; page 88

This was also highlighted by another participant, who emphasized that the AI4U training only set prompts, whereas actual processing and change had to be self-motivated:

No, I managed to [strengthen my skills and handle my emotions better in everyday life] on my own, but the app mainly helps in getting started with it and...become more aware of your emotions...So, before I can learn to deal with my emotions, I have to perceive them first. And the app helped with that. It didn’t help me so much with processing them, but processing mainly happens through the perception and then acceptance of my emotions.

PCI_P17; page 214

Another participant stated that he did not want to be controlled by an mHealth app and did not want an app to dictate a journey toward self-optimization:

If, for instance, it was an app that somehow had the message, “You have to become better or whatever,” then I would have dropped out of the study...I don’t want an app to dictate to me that things have to be better or whatever.

PCI_P11; pages 186-188

## Discussion

### Self-Reflection and Regulation of Negative Affect Improved During the AI4U Training

Our qualitative results point toward the positive impact of sampling emotions (via EMA) and real-time mobile interventions based on moment and life context (via EMI) on the mental health of young people. There was broad consensus across our participants that the EMA were helpful for self-reflection on emotions and their respective triggers. Previous qualitative studies have reported similar enhanced self-awareness among young people after engaging in EMA for up to 3 weeks [18,38,50,51]. Participants reported that the EMI embedded in the AI4U training improved emotion regulation and helped relativize negative emotions and unwind from daily challenges. Aligned with this, an existing mHealth app that provides digital cognitive reappraisal strategies to young people has resulted in reduced impulsivity among users [18]. Other mHealth apps have elicited similar positive effects on participants’ emotion regulation and reduction in stress or anxiety levels [12]. Our findings suggest that EMA may foster emotional awareness, which, in turn, seems to enhance the subjective effectiveness of EMI in managing and adapting to those emotions.

### Young People’s Trust in Disclosing Emotions on Digital Devices Is Driven by Anonymity

People’s trust is crucial for the willingness to share data and can be shaped by the perceived usefulness, sensitivity, and anonymity of personal health data [69]. As our participants tested the AI4U training as part of a research study and trusted the confidential data processing, they did not report any concerns regarding data security. For adequate trust in a digital intervention, qualitative studies highlight young people’s demand for transparent data handling underlying evidence-based approaches, credibility of the developers, and young people’s direct involvement through cocreation in the development process [46,70].

However, trust to disclose emotions was also driven by our participants’ perception of the mHealth app as nonhuman. According to them, the AI4U training provided a sense of emotional distance and anonymity compared to face-to-face communication and significantly facilitated the unveiling of emotions via EMA. Previous qualitative research has identified that anonymity on digital applications reduces young people’s fear of stigma associated with the disclosure of mental health issues [46]. There seems to be no significant difference in young people’s willingness to share data about mental health online compared to their willingness to disclose information about alcohol consumption, physical activity, or academic performance [71]. Our participants valued the AI4U training’s neutrality, especially when compared to the risk of judgment or potential misuse of data on mental health when interacting with friends, family, or mental health professionals. Our findings align with those of existing studies and suggest that discussing and sharing mental health issues on digital devices from scientific app providers is not a barrier for young people.

### Young People’s Flexible Engagement With mHealth Apps While Balancing Everyday Commitments

Some participants highlighted the prioritization of personal activities over AI4U training use when feeling well and resilient within their routines. In line with this, previous studies have identified a rather situational use pattern where an mHealth app is used only when immediate needs arise or in preparation for expected challenges [37,52]. In contrast, a few participants avoided using the AI4U training when experiencing extreme negative emotions. A previous study also identified high levels of overall negative affect among young people as negative contributors to their compliance rates with subsequent EMA prompts [72]. Our results suggest that there might be within-participant ambiguities in young people’s views on the training’s role in daily life. Some of our participants also acknowledged that it probably would have been most beneficial to engage with the mHealth app and follow the prompts during moments of emotional distress or psychological discomfort. Participants recognized that, without the AI4U training, they would not have allocated time for mental health–promoting activities in their daily life and noted that the AI4U training helped them become more aware of the need to take breaks, which they found difficult to implement without the training. In fact, it is tempting to speculate that EMI tailored to current needs, emotional state, and contextual factors may, in part, need to be perceived as disruptive to existing habits and daily life structures to nudge individuals and initiate change in psychological processes and mechanisms. In other words, timely interventions might be essential for achieving the intended health-promoting effects. To address this, future development should further strive to strike the right balance among minimizing perceived interference, optimizing acceptance, and ensuring that interventions are accessible and used during crucial moments when they can have the most impact.

Our findings suggest that young people tended to integrate the AI4U training into their existing daily routines and preferred to approach it in a flexible and nonbinding manner, that is, engaging with EMA or EMI depending on their personal capacity. Our participants perceived the AI4U training more as a “tool” used while maintaining user agency. Nonnegotiable commitments such as work or academic duties were central in their daily lives, and participants reported being rather reluctant to pause any of their ongoing activities to use the AI4U training. Time constraints and lack of alignment with other daily activities have previously been identified as major barriers to the use of mental health services among young people [6]. In line with previous research, young people tended to answer EMA questions rather superficially when being occupied with other tasks [51], and an increased stress level in young people is a predictor of noncompletion of subsequent EMA prompts [72]. According to some of our participants, it was difficult to integrate the AI4U training into their daily routines, and some felt stressed by the frequent alarms. However, the AI4U training already allowed for muting notifications, the interactions were rather brief (approximately 1 minute for EMA), and not every prompt included an EMI. Our participants were informed about the mute function during prestudy briefing sessions. Similarly, our research team emphasized during those briefing sessions that the training’s intention was not to interfere with daily life. Nevertheless, it seems that this did not result in all participants developing a relaxed attitude toward responding to the EMA or EMI or muting the notifications.

In another study, college students received up to 19 EMA per day but did not report feeling burdened by completing them [51]. This study used context- and signal-dependent prompts to avoid interrupting participants during specific levels of smartphone activity, for instance, if the data showed that they were in the process of sending an email. This approach may have contributed to young people feeling less overwhelmed. mHealth apps with gamification elements, a comprehensive backstory narrative, mini games, or performance levels have demonstrated lower attrition rates and enhanced resilience effects in young people compared to less gamified but equally evidence-based mHealth apps [73]. Image-based EMA prompts or prompts via wearable devices could also reduce use burden and enhance compliance among young people [51]. Future mHealth app development requires further participatory co-design to promote a continuous engagement of young people in mental health–promoting approaches.

Healthy young people may have found that their existing routines were effective and already helped them navigate their day-to-day activities, leaving little reason to change them. In contrast, those who are psychologically distressed may find that their coping mechanisms or routines hinder their daily lives. This experience might lead to greater openness toward the necessity of modifying these routines, for instance, through interventions such as EMI. In a previous cluster-randomized controlled trial, the absence of immediate and significant mental health–promoting effects among a nonclinical sample of healthy young people was attributed to their low baseline distress levels [19]. Thus, it is important to note that healthy people might report more disadvantages because they benefit less from the training.

### A Balance Between Self-Determination and Guidance in Daily App Use Behavior Stimulates Compliance

Some of our participants suggested that a greater self-directed use behavior could improve daily life integration, and some highlighted the value of an active involvement in the personalization process. In their development process into adulthood, young people pursue increased autonomy and independence, which also influences their mental health help-seeking behavior in daily life [9]. Their desire for shared decision-making approaches is in line with existing research on attitudes of young people toward mHealth apps. Previously, young people have stressed that an app should never replace human judgment and demanded an active role in steering AI decision-making processes or intervention and content selection [55,56,74]. This was introduced as the concept of “personalized personalization” [55]. Interviews with young people on other mHealth apps have shown that not the quantity but, rather, the timing of EMA throughout the day is challenging, and preset times of use would enhance adherence [38]. In the AI4U training, participants were allowed to set their activity times in advance and had the option to reschedule or trigger an EMA or EMI themselves if necessary. However, it seems that this flexibility was not clear to everyone. On the basis of those results, the option to choose a different EMI is now suggested more proactively in newer versions of the AI4U training. In addition, there is an increased emphasis on individual decision-making power in use behavior. It is important to note that, given the MRT design, only half of the EMI assignments in the AI4U training were AI based. Thus, this does not allow us to clearly draw conclusions about the quality of the AI decision-making processes.

A recent study cautions against increased customization by young people themselves given that “participants may express their desire for customizability in an interview situation, but in reality, they may respond better to clearer design, short interactions with the app, and easy access” [37]. This aligns with feedback from several participants, who reported difficulties in maintaining health-promoting routines learned during the study without the app, resulting in less frequent implementation or complete nonimplementation of these practices. In addition, some participants highlighted that excessive freedom of choice can be overwhelming, suggesting that AI can provide valuable support in this context.

The results point toward the important interplay between AI and human decision-making for an optimal user experience among young people. The challenge for future development processes of mHealth apps is to balance self-determination with targeted external guidance in use and decision-making to achieve the highest health-promoting outcome.

### Limitations

A major limitation of this study is the homogeneity of our study sample. Most of the interview participants (20/27, 74%) had obtained their high school diploma or were pursuing higher education. Participants in the PCIs and FGs were mainly students from disciplines including medicine, social work, business, and psychology. Thus, the recruitment strategy of this qualitative study reached predominantly young people who already actively engaged with mental or physical health and social behaviors in their everyday lives or academic education. Most students probably experienced higher levels of academic stress, and their strict daily schedules might have biased our results on daily life integration of the AI4U training. For students of such demanding academic fields, a high level of self-reliance is to be expected, which might also have influenced our results on demanded autonomy. Furthermore, we were unable to recruit young people from diverse migration backgrounds. Almost all the participants of this qualitative study (26/27, 96%) were born in Germany. Currently, there is limited research on the effects of mHealth apps on marginalized individuals from lower socioeconomic or with migration backgrounds [12,17,45]. Limited access to mHealth apps and a lack of alignment between digital interventions and the needs of marginalized individuals potentially limits their health-promoting effects and negatively influences compliance rates [75-77]. In addition, we recruited only a small number of participants, and the gender distribution was not evenly balanced. Due to low inclusion of young men in MRT 2, conducting a second FG with male individuals was not possible. Efforts to address data saturation by increasing the number of male participants in the PCIs did not yield the intended balance. A recent review on mHealth apps indicates a comparable skewness toward more female participants [17]. A potential explanation for this might be that help-seeking behavior is often associated with weakness [6,78]. There is a need for better understanding why particularly young men underuse health promotion services and what it takes to better engage this subgroup. One study, for instance, used a participatory approach to address gender differences in help seeking and mental health needs [79]. By adding gamification elements centering on sports (ie, football), they aimed for a more preference-based development process of an mHealth app particularly targeted at young men.

Some young people perceived the AI4U training as an additional stressor, with some participants highlighting that this stress was intensified by their desire to meet the requirements for adequate study participation. Young people also perceived the use of a second study smartphone for the AI4U training as burdensome. Hence, we cannot ascertain whether the stress stemmed from participation in the study or the app format itself. Due to the MRT study design, participants were unable to determine whether an EMI was based on random or AI-based assignment. Therefore, statements made in the interviews and FGs about the quality of the suggestions for EMI and the AI features must be interpreted with caution. We conducted the interviews and FGs with a subsample of all AI4U participants and only recruited participants for our qualitative study among the participants of MRT 1 and MRT 2. Therefore, generalizability of individual statements to the entire study population across all MRTs is limited.

### Conclusions

The approach of this study allowed for an in-depth understanding of young people’s experiences and views on the use of an AI-based mHealth app (AI4U training). Participatory approaches throughout the qualitative research process enabled the shared decision-making and empowerment of young people. The major contribution of our study is that, unlike previous qualitative studies that interviewed young people on their attitudes and preferences on mHealth apps or health promotion services [70,74], we elicited insights on real-world user experiences and subjectively experienced mental health–promoting effects. mHealth apps provide young people with an anonymous environment in which they can open up but that simultaneously allows for the maintenance of control over health-promoting actions. According to young people, an AI-based mHealth app can improve emotional resilience, self-awareness, and health-promoting routines in daily life. Our study also highlights some challenges with integrating an mHealth app into the structured routines of healthy young people. Therefore, flexibility and adaptability seem to enhance the reach, effectiveness, and scalability of an mHealth app.

## Appendix

Multimedia Appendix 1 AI4U training components.

Multimedia Appendix 2 Study participant overview.

Multimedia Appendix 3 COREQ (Consolidated Criteria for Reporting Qualitative Research) checklist.

Multimedia Appendix 4 AI4U script for the remote focus groups for the co-researchers.

Multimedia Appendix 5 Interview topic guide (problem-centered interviews and focus groups).

Multimedia Appendix 6 Coding categories (problem-centered interviews and focus groups).

## Abbreviations

AI: artificial intelligence
COREQ: Consolidated Criteria for Reporting Qualitative Research
EMA: ecological momentary assessments
EMI: ecological momentary interventions
FG: focus group
mHealth: mobile health
MRT: microrandomized trial
PCI: problem-centered interview
