# Supplementary material for: Health-Promoting Effects and Everyday Experiences With a Mental Health App Using Ecological Momentary Assessments and AI-Based Ecological Momentary Interventions Among Young People: Qualitative Interview and Focus Group Study
Source: JMIR Mhealth Uhealth. 2025 Apr 29;13:e65106. doi: 10.2196/65106 (PMC12076033; doi:10.2196/65106)
Supplement: Multimedia Appendix 1 [file mhealth_v13i1e65106_app1.docx]

| **Ecological Momentary Intervention (EMI)** | **Short description** |
| --- | --- |
| ***Emotional Compass*** | The emotional compass consists of three emotional domains: (1) danger, (2) calm and security, and (3) goals and needs. Users learn to better understand and classify their emotions and how the three domains influence their perception and emotions |
| ***Breathing Intervention*** | Teaches how to find a soothing and beneficial breathing rhythm that strengthens calmness and the security system |
| ***My Quiet and Safe Place*** | Visualization interventions that helps participants find a calming and secure mental space, encouraging them to imagine a place that soothes them for 2 minutes |
| ***Empathetic Companion*** | Guides users to visualize a personal compassionate companion, free to take any form, who understands their experiences and offers non-judgmental support in challenging situations |
| ***Emotions as Wave*** | Introduces the idea of viewing emotions as ocean waves that come and go, emphasizing the importance of riding these waves instead of being overwhelmed by them |
| ***Moments of Joy*** | Participants can record personal moments of happiness in the app, creating a growing diary-like list, to focus attention on positive aspects of daily life |
| ***Positive Data Log*** | Participants maintain a diary of daily small or significant achievements to focus on their successes, fostering self-efficacy and self-confidence |
| ***Activity Planner*** | Participants create a daily schedule of activities from different life domains to improve mood and monitor their progress |

| **EMA Item no.** | **Conditional items** | | **EMA Item** | **Visibility rule** | **EMA Name** | **Response** | **Target construct** |
| --- | --- | --- | --- | --- | --- | --- | --- |
| 1 |  |  | This is the first EMA I am completing today. |  | EMA_firstsignal | binary | - |
|  | 1.1 |  | I have slept well last night. | If item no. 1. --> 1 ("Yes") | EMA_sleep | 1-7 | Sleep |
|  | 1.2 |  | I am excited about the day. | If item no. 1. --> 1 ("Yes") | EMA_joyful_day | 1-7 | Quality of life |
| 2 |  |  | I feel good. |  | EMA_mood | 1-7 | Positive affect |
| 3 |  |  | I am disappointed in myself. |  | EMA_disappointed | 1-7 | Self-esteem |
| 4 |  |  | I feel afraid. |  | EMA_scared | 1-7 | Negative affect |
| 5 |  |  | I am thinking about my problems. |  | EMA_worry | 1-7 | Worrying |
| 6 |  |  | I feel downhearted. |  | EMA_down | 1-7 | Negative affect |
| 7 |  |  | I feel self-confident. |  | EMA_confidence | 1-7 | Self-esteem |
| 8 |  |  | I feel sad. |  | EMA_sad | 1-7 | Negative affect |
| 9 |  |  | I feel relaxed. |  | EMA_relaxed | 1-7 | Positive affect |
| 10 |  |  | I feel energetic. |  | EMA_energetic | 1-7 | Activity level |
| 11 |  |  | I feel stressed. |  | EMA_stress | 1-7 | Stress |
| 12 |  |  | I am satisfied. |  | EMA_satisfied | 1-7 | Positive affect |
| 13 |  |  | I have my negative feelings under control. | If item no. 2 = rated <3 // 4 or 6 or 8 = rated >4 | EMA_emotion_control | 1-7 | Emotion regulation |
| 14 |  |  | I feel lonely. |  | EMA_lonely | 1-7 | Social isolation |
| 15 |  |  | I have difficulties concentrating. |  | EMA_concentration | 1-7 | Stress / concentration |
| 16 |  |  | I am tired. |  | EMA_tired | 1-7 | Activity level |
| 17 |  |  | I can change my negative feelings to positive ones. | If item no. 2 = rated <3 // 4 or 6 or 8 = rated >4 | EMA_emotion_change | 1-7 | Emotion regulation |
| 18 |  |  | These complaints, thoughts, or feelings are occupying me the most right now (optional): |  | EMA_heaviestcomplaint | open text | - |
| 19 |  |  | I have been physically active since the last EMA. |  | EMA_feelactive_sincebeep | 1-7 | Activity level |
| 20 |  |  | I can handle any difficulties I might encounter. |  | EMA_resilience | 1-7 | Resilience |
| 21 |  |  | This is what I am doing right now: |  | EMA_activity_current | categorical | - |
|  | 21.1 |  | What other activity/activities? | If item no. 21 --> 14 ("Other") | EMA_activity_other | open text | - |
|  | 21.2 |  | What I am doing right now is pleasant. |  | EMA_activity_pleas | -3 / +3 | Stress |
| 22 |  |  | I am alone. |  | EMA_social_alone_yes | binary | - |
|  | 22.1 |  | Who am I with? | If item no. 22 --> 2 ("No") | EMA_social | categorical | - |
|  | 22.2 |  | This is pleasant. | If item no. 22 --> 2 ("No") | EMA_social_pleas | -3 / +3 | Stress |
|  | 22.3 |  | I would rather be with people. | If item no. 22 --> 1 ("Yes") | EMA_company | binary | Stress |

| **Proximal outcomes - EMAs** |
| --- |
| Positive affect (3 items) - primary |
| Negative affect (3 items) - secondary |
| Stress (3 items bzw. 4) - secondary |
| Resilience (1 item) - secondary |
| Emotion regulation (2 items) - secondary |
| Self-esteem (2 items) - secondary |
| Activity level (3 items) - secondary |
