# Supplementary material for: Health-Promoting Effects and Everyday Experiences With a Mental Health App Using Ecological Momentary Assessments and AI-Based Ecological Momentary Interventions Among Young People: Qualitative Interview and Focus Group Study
Source: JMIR Mhealth Uhealth. 2025 Apr 29;13:e65106. doi: 10.2196/65106 (PMC12076033; doi:10.2196/65106)
Supplement: Multimedia Appendix 2 [file mhealth_v13i1e65106_app2.docx]

| **Problem-centered interviews (N= 16)** | | | | | | |
| --- | --- | --- | --- | --- | --- | --- |
| **Participant** | **MRT** | **Interviewer^[[1]](#footnote-1)^; Duration** | **Age** | **Gender** | **Educational level^[[2]](#footnote-2)^** | **Migration background^[[3]](#footnote-3)^** |
| PCI_P11 | 1 | SH; 41:21 | 24 | male | High school diploma | - Father: Austria - Grandparents paternal side: Austria; maternal side: USA/Germany |
| PCI_P12 | 1 | SH; 58:00 | 18 | female | Intermediate secondary school | - Parents: Both Kosovo - Grandparents: Both Kosovo |
| PCI_P13 | 1 | SH; 51:18 | 24 | female | High school diploma | - Parents: Both Pakistan - Grandparents: Both Pakistan |
| PCI_P14 | 1 | SH; 55:03 | 22 | female | Bachelor´s degree | No |
| PCI_P15 | 1 | CG; 47:30 | 18 | male | Intermediate secondary school | No |
| PCI_P16 | 1 | SH; 58:20 | 18 | male | Intermediate secondary school | No |
| PCI_P17 | 1 | SH; 01:10:57 | 25 | male | High school diploma | - Grandparents maternal side: Germany/Czech Republic |
| PCI_P21 | 2 | SH; 39:22 | 14 | male | Secondary General school; finished without certification | - Grandparents maternal side: Germany/Czech Republic |
| PCI_P22 | 2 | SH; 36:24 | 25 | male | Bachelor´s degree | No |
| PCI_P23 | 2 | SH; 40:49 | 21 | female | High school diploma | - Mother: Poland - Grandparents maternal side: Both Poland |
| PCI_P24 | 2 | CG; 30:00 | 21 | female | High school diploma | No |
| PCI_P25 | 2 | CG; 31:19 | 23 | male | High school diploma | No |
| PCI_P26 | 2 | SH; 42:51 | 20 | female | High school diploma | No |
| PCI_P27 | 2 | CG, 35:12 | 15 | female | Secondary General School | No |
| PCI_P28 | 2 | SH, 32:40 | 16 | female | Intermediate secondary school | No |
| PCI_P29 | 2 | SH, 46:40 | 22 | male | High school diploma | - Grandparents parental side: Germany/Austria |

| **Focus Groups (N=11)** | | | | | | | |
| --- | --- | --- | --- | --- | --- | --- | --- |
| **Participants** | **MRT** | **Group** | **Interviewer^[[4]](#footnote-4)^, Duration** | **Age** | **Gender** | **Educational level** | **Migration background** |
| FG1_P1 | 1 | 1 | Moderation (JS);  Co-Moderation (CG); 01:04:23 | 21 | female | High school diploma | - Parents: mother Poland - Grandparents maternal side: Poland |
| FG1_P2 | 1 | 1 |  | 25 | female | High school diploma | - Parents: father Turkey - Grandparents parental side: Turkey |
| FG1_P3 | 1 | 1 |  | 18 | female | Intermediate secondary school | - Parents: father Spain - Grandparents parental side: Germany/Spain |
| FG2_P1 | 1 | 2 | Moderation (SD);  Co-Moderation (SH);  01:03:43 | 21 | male | High school diploma | - Grandparents maternal side Poland/Unknown; parental side France/Germany |
| FG2_P2 | 1 | 2 |  | 22 | male | High school diploma | - Grandparents maternal side Germany/Serbia; parental side Serbia/Czech Republic |
| FG2_P3 | 1 | 2 |  | 23 | male | High school diploma | - Grandparents maternal side unknown; parental side Poland/Germany |
| FG3_P1 | 2 | 1 | Moderation (JS); Co-Moderation (CG);  01:26:18 | 21 | female | High school diploma | - born in GB, - Father: Netherlands - Grandparents parental side: Netherlands |
| FG3_P2 | 2 | 1 |  | 22 | female | High school diploma | No |
| FG3_P3 | 2 | 1 |  | 19 | female | High school diploma | - Parents: Father Iraq, Mother Libya - Grandparents parental side Iraq; maternal side Libya |
| FG3_P4 | 2 | 1 |  | 22 | female | High school diploma | - Mother: Turkey - Grandparents maternal side: Turkey |
| FG3_P5 | 2 | 1 |  | 21 | female | High school diploma | No |

1. Selina Hiller (SH). Christian Goetzl (CG). [↑](#footnote-ref-1)
2. This inquiry requested the highest educational achievement attained. Please note that for instance some respondents may currently be studying, even if we state "high school diploma" here. Only completed educational levels are indicated. High school diploma stands for the German “Abitur”. Intermediate secondary school stands for the German “Realschulabschluss”. Secondary General School stands for German “Hauptschulabschluss” [↑](#footnote-ref-2)
3. Migration background was requested on three levels: 1.) the participants country of birth, 2.) the parents’ country of birth, 3.) maternal and paternal grandparents’ country of birth. “No” indicates that the participant him-/herself and the (grand-)parents were all born in Germany. [↑](#footnote-ref-3)
4. For focus groups, moderation was conducted by two of our Co-Researchers from the participatory research workshops. Co-Moderators were research team members and supported the Co-Researchers in conducting the focus groups. Julia Sauter (JS). Sina Dietrich (SD). [↑](#footnote-ref-4)
