# Supplementary material for: Health-Promoting Effects and Everyday Experiences With a Mental Health App Using Ecological Momentary Assessments and AI-Based Ecological Momentary Interventions Among Young People: Qualitative Interview and Focus Group Study
Source: JMIR Mhealth Uhealth. 2025 Apr 29;13:e65106. doi: 10.2196/65106 (PMC12076033; doi:10.2196/65106)
Supplement: Multimedia Appendix 4 [file mhealth_v13i1e65106_app4.docx]

**Guideline for remote data collection**

**15 minutes before the focus group starts - Open the Zoom room and answer questions**

[Moderator & Co-Moderator present]

- Provide technical assistance if needed / verify working connections
- If disruptions occur, suggest individual participants to use headphones
- Point out names if someone has logged in with their full name
- Postpone substantive questions to the beginning of the focus group

**At the start of the focus group**

- Greet all participants
- Can everyone hear me? Please give me a quick thumbs-up if that's the case!
- My name is XY. I am a research assistant at the University of Ulm and today I'm here as a co-moderator. I'll support the main moderator a bit with technical issues, occasionally ask questions, and make sure everyone gets a chance to participate.
- There will be a brief introduction round soon. Here, moderator XX will also introduce herself and share something about her.

But before that, a couple of basic things:

- Is everything working fine with the technology, and does everyone know how to unmute themselves, etc.?
- In video conferences, it's usually best to mute when not speaking. However, since we want to have a discussion, let's try without muting first. We'll test during the introduction round for any issues.
- For those wearing headsets or headphones: that's very helpful. Thank you! If others also have headsets/earphones, it would be helpful if you could connect those shortly.

A brief overview of the procedure:

- The focus group will last about 90 minutes and will be recorded by us. Later, it will be transcribed, and all names, locations, etc., will be removed. Everything you say stays within this virtual space and cannot be traced back to you.
- Your thoughts will greatly assist us today. There are no right or wrong answers. We're interested in your opinions.
- If you have any questions, please let us know at any time. Participation is voluntary! You're free to leave anytime. But of course, we hope you stay until the end.
- To speak, you can simply unmute and talk. Or you can also use the "raise hand" function, whichever you prefer.
- If there are further discussions or questions after the focus group, we'll keep the video chat open for about 5 minutes after the discussion ends.
- Are there any general questions about the study before I start the recording and XY takes over the moderation?

[Wait for reactions from participants]

Then I will start the recording now!

[Check if the recording is running]

Then [Moderator], you can start the moderation.

**[Moderator continues with the interview topic guide!]**

**-----------------**

- End of the feedback round -

[Now I will stop the recording]

That's all from us, a heartfelt thank you for your participation and time. Your experiences greatly assist us, and we'll try to adjust the AI4U project to your ideas and recommendations during the development phase.

You will receive the vouchers for participation in an email tomorrow at the latest!

Feel free to stay for any questions/discussions! Otherwise, I wish you a pleasant evening and all the best!
