# Supplementary material for: Health-Promoting Effects and Everyday Experiences With a Mental Health App Using Ecological Momentary Assessments and AI-Based Ecological Momentary Interventions Among Young People: Qualitative Interview and Focus Group Study
Source: JMIR Mhealth Uhealth. 2025 Apr 29;13:e65106. doi: 10.2196/65106 (PMC12076033; doi:10.2196/65106)
Supplement: Multimedia Appendix 5 [file mhealth_v13i1e65106_app5.docx]

**Interview Topic Guide: Problem-centred interviews^[[1]](#footnote-1)^**

**MRT 1**

**October 2022 – March 2023**

| **Narrative / Guiding question** | **Follow-up questions** | **Content focus** |
| --- | --- | --- |
| 1. **Introduction** |  |  |
| Firstly, we're curious about your overall experience with using the app. How was it for you? |  | **Introductory question on first impression** |
| 1. **App usage in general / in everyday life** |  |  |
| And what did a typical day with the app look like for you?  *(If applicable: Could you describe a day with the app for me?)* | How was it throughout the day? How can I imagine that? | **Description of a typical day with the app** |
| How would you say, what importance did the app have in your daily life? | How successful was it to integrate the app into your daily life?   - Why? Why not? - Could you provide me with an example?   How important or unimportant has it been in your daily life?   - Why? Why not? - Could you describe that in more detail? (optional) | **Integration of the app into everyday life**  **Importance of AI4U-Training** |
| How did you handle the interventions or mood queries? To what extent did you, for instance, always complete them when suggested by the app? | So, how was it for you to receive a recommendation to do an intervention in a particular situation?  Did you ever dismiss it? How often? Why?   - Temporal aspects (challenging?) | **Handling EMA and EMI** |
| 1. **Mood assessments / EMA** |  |  |
| You received mood assessments several times a day through the app, asking about your current feelings and the situation you were in.  How did you generally feel about these mood assessments? | Have you ever skipped responding to the EMA? How often did you do so, and what was the reason behind it?  How did you feel about expressing your feelings to the AI4U app? | **General evaluation and handling of EMA**  **Expression of emotions** |
| What did you think of the questions and possible answers you were asked? | Were the questions and possible answers formulated sensibly in your opinion?  Do you think the app managed to understand your mood correctly/capture it well?   - Why, why not? - Could you provide me with an example? | **Evaluation of EMA questions/answers**  **Fit of EMA to emotions** |
| Were the questions and answer options appropriate for the respective situation? | Did the questions/answer options cover everything you would have liked to tell the app? (e.g. questions about relationship/partnership?) |  |
| Did you have the impression that some of the questions were personal or intimate? | How did you perceive that?  How did you deal with that? | **Sharing emotions / Intimacy** |
| Who else do you talk to about your moods or feelings in everyday life? | Has the app changed who or what you talk to about your moods and feelings? | **Talking about feelings in everyday life**  **Change through the app** |
| 1. **Evaluation of the Artificial Intelligence** |  |  |
| As you know, the AI4U-Training app works with artificial intelligence (AI for short). Have you noticed this while using it? | How did you notice that?  Do you think this improved usage or motivated you more? | **Evaluation of AI aspects** |
| What did you think of the selection of interventions the app suggested to you? | Were you happy with it?   - Content-wise, were suitable exercises suggested to you? - Timing-wise, did they come at the right moment when you also wished for support? | **Fit of selected EMI to emotions** |
| 1. **Interventions / EMI and the training effect** |  |  |
| Is there anything that using the app has changed for you? | Can you say something more about this?  How do you determine this? | **Changing habits** |
| Which of the exercises helped/benefited you the most?  *[Input: Show a screenshot of interventions on the slide]* | Why?  How did you notice that an exercise was helpful for you? (Optional: Has this changed your emotional state?) | **Examination of health promoting effect of interventions** |
| Which of the exercises helped/benefited you less?  Would you say that some exercises were too easy or too difficult? | How did you notice that an exercise was less helpful for you?  How often did you skip the exercises? Why? | **Handling EMI** |
| Are there any topics or exercises that you missed from the AI4U training? | e.g. solving conflicts in relationships/partnerships, the topic of sleep | **Missing topics** |
| 1. **Personal monitoring / dashboard** |  |  |
| Another aspect of the AI4U app is the dashboard, which provides an overview of all mood assessments.  *[Input: Show dummy dashboard on the web browser]*  We would like to know: Have you used the dashboard? And if so, how did you use it? | What was that like for you?  Can you say more about that? | **General usage of dashboard function** |
| How do you find the dashboard? (e.g. structure) | How important were the results for you?  How was that for you?  Was that helpful for you? Why? Why not? | **Importance of dashabord results** |
| How would you rate the dashboard? Did it help you? | Did you notice any correlations or changes over time on the dashboard?  Could you provide an example? | **Changes in the dashboard** |
| **[note: for participants who had additional face-to-face counseling sessions only]**  You mentioned earlier that you had a discussion about the dashboard with a consultant. How did you find that discussion?  Can you describe how it went?  Did the consultant provide you with a different perspective on the results? | Did you feel comfortable/uncomfortable? Too personal/intimate? | **Reflection of participatory consultation appointments** |
| 1. **Sustainable effects of use** |  |  |
| Would you say that using the app changed your habits or daily routine?  (For example, because you performed an exercise recommended by the app that should have been helpful for you at that moment) | Will you continue to implement what the app suggested or advised?  Do you believe you'll continue doing the exercises even without the app?   - What might be challenging about this, or what would you need to continue doing them? | **Long-term effects of app usage / Sustaining the benefits** |
| Do you think such an app could be a long-term companion? | Why? Why not?  What makes you think so? | **Long-term usage behavior** |
| You completed the AI4U-Training for a total of 40 days. Would you say that through using the app, you've learned or discovered something about yourself? | Have you possibly learned something about yourself and your well-being that you didn't know before?   - For instance, which situations are stressful for you?   Do you think the app helped you better cope with your daily challenges?   - For example, being able to relax more easily after an exam? | **Learning outcomes**  *[Note to the interviewer: This part concerns personal health-related risk/behavior factors]*  **Resilience outcomes** |
| The AI4U-Training aims to strengthen your ability to better cope with emotions and stress in daily life.  How do you perceive this goal?   - Would you say it has achieved this? | How did you notice that? | **Expectations and demands towards a mHealth app** |
| 1. **Resume and closing** |  |  |
| I've learned a lot from you. Is there anything else you'd like to share that we haven't discussed yet? | Should we pass something on to our colleagues in the programming/design of the app? | **Open discussion points / improvement implications** |

Thank you for your participation, your ideas will contribute to the further development of the AI4U-Training app.

**Interview Topic Guide: Problem-centered Interviews^[[2]](#footnote-2)^**

**MRT 2**

**July – November 2023**

| **Narrative / Guiding question** | **Follow-up questions** | **Content focus** |
| --- | --- | --- |
| 1. **Introduction** |  |  |
| Since the app is intended to fit well into the daily lives of the people who use it, I'd like to start by learning a bit more about you and your daily routine.  Would you like to **introduce yourself** first and share a bit about who you are and what you usually do? | How does your daily routine look right now? Could you describe a typical day in your life? | **Introductory question**  **Description of usual daily life** |
| 1. **App usage in general / in everyday life** |  |  |
| Now I would like to learn more about your experience using the AI4U app in your daily life.  When you first used the AI4U app, do you remember how you felt about it and what thoughts you had? | What were your expectations for the app? | **First impressions**  **Expectations** |
| And what did a typical day with the app look like for you? | Could you describe a day with the app for me? | **Description of a typical day with the app** |
| How did you integrate the app into your daily life? | *(Note: Let the participant describe the usage)*  Where was it easy, and where was it a bit more challenging?  Could you provide me with an example? | **Integration of the app into everyday life** |
| 1. **Mood assessments / EMA** |  |  |
| You received mood assessments several times a day through the app, asking about your current feelings and the situation you were in. I have a summary about this and would like to share my screen briefly for that.  *[Input: Show a screenshot of exemplary mood assessments]*  How did you generally feel about these mood assessments?  What role did the mood assessments play in your daily life? | How did you feel about expressing your feelings to the AI4U app?  Were there situations where you particularly liked or disliked answering the mood assessments?  Have you ever skipped responding to the EMA? How often did you do so, and what was the reason behind it? | **Perspectives / opinion on mood assessments / EMA**  **General evaluation of EMA / Expressing emotions**  **Integration in daily life**  **Handling mood queries** |
| How accurately do you feel the app captured your mood?  (*Explanation: Did the app understand HOW you were feeling?*) | Why, why not?  Could you provide me with an example? | **Fit of EMA to emotions** |
| Did the questions/answer options cover everything you would have liked to tell the app? | Were you also missing topics or questions?  (e.g. questions about relationships/partnerships?) | **Sharing emotions** |
| Who else do you talk to about your moods or feelings in everyday life?  Has the app changed anything about it? |  | **Talking about feelings in everyday life**  **Change through the app** |
| 1. **Interventions / EMI** |  |  |
| Besides mood assessments, you also received several exercise suggestions throughout the day. I have a summary here to show you.  *[Input: Show a screenshot of interventions on the slide]*  In general, how did you find the interventions?  Do you think the app succeeded in offering you the appropriate exercise at the right time? | Were there situations where you particularly liked or disliked conducting an intervention?  Have you ever skipped an EMI? How often did you do so, and what was the reason behind it?  Timing-wise, did they come at the right moment when you also wished for support?  Why? Why not?  Could you provide me with an example? | **Perspectives / opinion on interventions / EMI**  **General inquiry about exercise**  **Handling interventions**  **Fit of EMI to emotions** |
| 1. **Personal monitoring / dashboard** |  |  |
| Another aspect of the AI4U app is the dashboard, which provides an overview of all mood assessments.  *[Input: Show dummy dashboard on the web browser]*  Did you use the dashboard? | How was that for you?  Was that helpful for you? Why? Why not?  How important were the results for you? | **General usage of dashboard function**  **Importance of results** |
| Did you notice any correlations or changes over time on the dashboard? | Can you provide an example? For instance, any instances where your mood dips aligned with mood surveys? | **Changes in the dashboard** |
| **[note: for participants who had additional face-to-face counseling sessions only]**  You mentioned earlier that you had a discussion about the dashboard with a consultant. How did you find that discussion?  Can you describe how it went?  Did the consultant provide you with a different perspective on the results? | What was that like for you?  How did you perceive that?  Could you describe a typical process of such an appointment for me? | **Reflection of participatory consultation appointments** |
| 1. **Subjective effects / benefits of the app** |  |  |
| How would you describe the effect of the app, what did it achieve for you? | How did you notice that an intervention was helpful for you?  Can you recall interventions that were particularly beneficial for you, or perhaps some that were less helpful or effective? | **Overall effectiveness**  **Examination of health promoting effect of interventions** |
| To what extent have your habits or everyday life changed through using the app? | Where was that? | **Changing habits** |
| Is there anything you learned from using the app, perhaps also learned about yourself? | Have you perhaps learned something about yourself that you didn't know before? | **Learning outcomes** |
| 1. **Trust** |  |  |
| Were you able to trust the AI4U-Training app? | Explanation if nothing happens:  So generally trust in terms of what data the app gets from you and what information you disclose  *(Note: The additional request is rather optional, regarding keywords: data protection, security..)* | **Trust into the app** |
| 1. **Sustainable effects of use** |  |  |
| What's next, so now regarding the mood assessments and intervention - will you continue to implement what the app suggested or advised? | … also withouth the app? | **Long-term effects of app usage / Sustaining the benefits** |
| Could you envision using the app permanently? | What would be an ideal duration for using the app in your opinion, and why?  (e.g. from a few days to permanently) | **Usage duration beyond the official study period** |
| 1. **Resume and closing** |  |  |
| You talked about your initial expectations at the beginning. Would you say that your expectations of the app have been met? |  | **Fulfilled / unfilled expectations** |
| I've learned a lot from you. Is there anything else you'd like to share that we haven't discussed yet? |  | **Open discussion points** |

Thank you for your participation, your ideas will contribute to the further development of the AI4U-Training app.

**Interview Topic Guide: Focus groups^[[3]](#footnote-3)^**

**MRT 1: December 2022 and February 2023**

**MRT 2: July 2023**

| **Narrative / Guiding question** | **Follow-up questions** | **Content focus** |
| --- | --- | --- |
| 1. **Introduction** |  |  |
| Hello from my side too, I'm [*name of Co-Researcher*], [*XX*] years old and will be moderating the focus group today.  I'm going to ask a variety of questions today and as [*Co-moderator, i.e. research assistant*] said, there are no right or wrong answers to these questions. You also don't have to answer anything if you are unsure whether you want to answer it (e.g. because the question is too personal for you). We just want to find out a little bit from you about how it worked with the app, what it was like in everyday life and what could be improved.  Just for information about the group that is here today: everyone here has already used the app for 40 days. This means that you are all already familiar with how to use the app and have gained experience here. A small difference may be that some tried the app for our study together with a professional (e.g. a school psychologist or other counselor) and others used it alone.  In order to warm up to each other as a group, it would be nice if we could all briefly introduce ourselves by our first names and, if you would like, briefly say something about yourself. E.g. how old you are, why you might have wanted to take part today or simply what you would like to say.  [*Respective co-moderator, i.e. research assistant]* do you start and then call someone? | [Eventual follow-up question]  z.B. May I ask how old you are? | **Introduction of participants** |
| 1. **App usage in general / in everyday life** |  |  |
| Thanks for the introduction. Yes, you all have been using the AI4U training for some time now. Before we go into the individual components of the app:  How did you generally get along with the app? | [First ask superficially:]  How clear was the app for you (structure?)  Did you have any technical problems using the app?  What was it like for you to use the app with a study cell phone and not with your private smartphone? | **Functionality in everyday life**  **Technical aspects**  **Aspects of study design** |
| Was it easy to integrate the app into your everyday life?  ​ | Did you have enough time to complete the surveys and exercises?  What would have been necessary to make the app more compatible with your everyday life? | **Integration of the app into everyday life**  **Improvement implications for everyday life integration** |
| Were the questions asked at the right time?  Were the interventions suggested at the right moment? | How did you find the number of daily EMA/EMI? | **Fit of EMA / EMI to emotions and preferences** |
| 1. **Interventions / EMI** |  |  |
| Wir würden nun näher auf die Trainings*inhalte* eingehen wollen. Hierzu haben wir eine Folie mit einer Übersicht des Trainingsspektrums vorbereitet.  ***[ppt. slide 🡪 Co-Moderation: share screen]*** | What did you think of the individual exercises?  [*note: go through all exercises*]  Did you find the exercises easy to implement?  How diverse were they? Were you missing or bothered by any exercises in the app? | **Perspectives / opinion on interventions / EMI**  **Ease of use**  **Intervention range** |
| 1. **Personal monitoring / dashboard** |  |  |
| Another aspect of the AI4U app is the dashboard, which provides an overview of all mood assessments.  ***[ppt. slide 🡪 Co-Moderation: share screen]***  We would first like to know: Have you ever used the dashboard? | ***[note:* for participants who had additional face-to-face counseling sessions only*]***  For those who used the dashboard with a professional (consultant): Did you always use it together with the professional or sometimes alone? | **General usage of dashboard function**  **Usage behavior of participants who had additional counselling sessions** |
| What did you think of the dashboard? | How often have you looked at your results online?  Was this helpful for you?  Is there anything about the dashboard that you would like to change?  Who else do you talk to about your moods/feelings? With friends? Your parents? | **Importance and effectiveness of results**  **Improvement implications**  **Talking about feelings in everyday life** |
| ***[note: only if participants who had additional face-to-face counseling sessions are in the focus groups and if they feel comfortable revealing that they had those additional sessions]***  If you've tried dashboard discussion with a professional, what did you think of the consultation appointments? | Was this helpful for you?  Was it strange or perhaps even better to do it together with a professional?  Was the professional able to give another perspective on the results?  Was that too personal/intimate for you? | **Reflection of participatory consultation appointments** |
| 1. **Evaluation of the Artificial Intelligence** |  |  |
| The special thing about the AI4U app is that an artificial intelligence (AI) selects the interventions based on your previous use of the app.  How did you find the selection of exercises?  How did you notice that the app was controlled by an AI? | Did you feel that the suggested exercises were a good fit for your current state of mind?  Have you ever been surprised at how well the app's suggestions suited you? | **Fit of EMI to emotions**  **Evaluation of AI in the intervention selection** |
| 1. **Trust** |  |  |
| Did you feel comfortable that an AI worked with your information? | What do you think about AI collecting data from you? | **Data privacy and trust** |
| Why would you stop using an AI-informed app? | If you have already used mHealth apps without AI: Have you noticed a difference with the AI4U app? | **Usage barriers** |
| 1. **Resume and closing** |  |  |
| What did you think was good about the app overall? What did you think was bad about the app overall? | What would you change about the app?  (if you could change anything about it?) | **Overall evaluation of the app** |
| Would you recommend the app to your best friend?   - Why why not? | How would you see the overall benefit of the app now? | **Experienced benefit and recommendation to friends** |
| Do you think the app is for everyone or perhaps only for specific individuals? | e.g., tech enthusiasts, noting for boys, etc.?  What would have to be different so that those people would also use the app? | **Potential dialogue population** |
| Now we have learned a lot from you. Do you have any comments or topics that we haven't talked about yet but that are important to you? |  | **Closing question** |

Thank you for your participation, your ideas will contribute to the further development of the AI4U-Training app.

1. In square brackets there are instructions to the moderators, e.g. “[First ask superficially:]”, or [ppt. Slide 🡪 share screen], in round brackets there are partial aspects of individual questions that can possibly be taken up in a follow-up question: e.g. “How clear was the app for you (structure?)” [↑](#footnote-ref-1)
2. In square brackets there are instructions to the moderators, e.g. “[First ask superficially:]”, or [ppt. Slide 🡪 share screen], in round brackets there are partial aspects of individual questions that can possibly be taken up in a follow-up question: e.g. “How clear was the app for you (structure?)” [↑](#footnote-ref-2)
3. In square brackets there are instructions to the moderators, e.g. “[First ask superficially:]”, or [ppt. Slide 🡪 share screen], in round brackets there are partial aspects of individual questions that can possibly be taken up in a follow-up question: e.g. “How clear was the app for you (structure?)” [↑](#footnote-ref-3)
