# Supplementary material for: Health-Promoting Effects and Everyday Experiences With a Mental Health App Using Ecological Momentary Assessments and AI-Based Ecological Momentary Interventions Among Young People: Qualitative Interview and Focus Group Study
Source: JMIR Mhealth Uhealth. 2025 Apr 29;13:e65106. doi: 10.2196/65106 (PMC12076033; doi:10.2196/65106)
Supplement: Multimedia Appendix 6 [file mhealth_v13i1e65106_app6.docx]

Coding tree of problem-centred interviews^[[1]](#footnote-1),^^[[2]](#footnote-2)^

| **Thematic (sub-)categories** | **Codes** | **Subcodes** |
| --- | --- | --- |
| **0. Socio-demographic data** | - | - |
| **1. Description of daily life (general, without app)** | - | - |
| **2. Expectations towards the app/AI4U training** |  |  |
| **2.1 Expectations beforehand** | I already knew what was coming because my friend had already used it |  |
|  | It’s exciting that AI and mental health come together |  |
|  | App for stress-reduction |  |
|  | App for mood monitoring/reflection |  |
|  | Afraid it could be too annoying |  |
|  | I had no expectations in advance |  |
|  | App as everyday assistant |  |
| **2.2 Expectations afterwards** | The app should be more of a structuring everyday helper |  |
|  | Will probably not use it permanently |  |
|  | Not a perfect fit for a 'healthy' dialogue population |  |
|  | Enhance gamification / design | Vs. risk of being too gamified, risk of focusing on level achievement |
|  | Content became boring | Variety must remain |
|  | Expectations were fulfilled | Surprised that using the app wasn't overwhelming/annoying |
|  |  | The structure of the EMI were surprisingly positive |
| **3. Integration of the app into everyday life** |  |  |
| **3.1. Integration in daily life** |  |  |
| **3.1.1 Good integration** | The app had to fit into the my daily routine |  |
|  | Tried not to skip EMA/EMI often |  |
|  | Usage routine was established | less annoying once it became a routine |
|  | Length of EMI is feasible to integrate in daily life |  |
|  | I missed the AI4U training after the end of the study |  |
|  | I found good timepoints for EMA |  |
| **3.1.2 Potential for improvement/ examples of poor integration** | EMA timepoints were sometime inappropriate | “Success Diary” too early in the day |
|  | Difficult to integrate into everyday life | too many EMA per day |
|  |  | time-consuming |
|  |  | vs. has “merged” into everyday life |
|  | Unusual at first | EMA initially “weird” (especially for introverted persons) |
|  |  | Change: Smartphone that asks how you are doing |
|  | Second study smartphone was problematic | was annoying |
|  |  | difficult to carry two phones (private and study smartphone) |
|  |  | Smartphone was forgotten somewhere |
|  |  | Therefore, I missed the EMI/EMA |
|  | Demand for self-determined usage behavior | Feedback at the end of the EMI |
|  |  | Setting priorities for content selection |
|  |  | Control over the duration/extent of the EMI |
|  |  | More control over timing/frequency |
|  |  | Postponing EMA/EMI not flexible enough |
|  |  | Schedule: Overview of number and timepoint of EMA/EMI helpful |
|  |  | Pausing for a longer period of time would be helpful |
|  |  | It should also be possible to postpone the EMI further |
|  | Vs. It was „enough“ self-determination | Postponing-feature was flexible enough |
|  | Preference for interpersonal contact during leisure time | Only when you have nothing to do |
|  |  | “I had other things to do” |
|  |  | Minor added value to existing personal relationships |
|  |  | “My Quiet and Safe Place” is already at home |
|  |  | I already have „My Empathetic Companion“ |
|  |  | Don't want to pause activities for the app |
|  |  | Example: When you have a moment of joy/success, you don't immediately take out your smartphone |
|  |  | vs. used with friends, if they were interested |
|  | No app usage, when in social settings / surrounded by people | Had to justify smartphone use to friends |
|  |  | more difficult in front of other people |
|  |  | Lack of concentration / too much distraction |
|  |  | Worry that smartphone usage is unfriendly |
|  |  | “uncomfortable” in front of other people |
|  |  | Uncomfortable when others see EMA entries (especially negative emotions) |
|  |  | Worried about how conducting an EMI looks like from the outside |
|  | Distracted from other things | You would have had to pause other activities |
|  | Would be easier with a self-determined daily life | Easily feasible at home, no additional burden |
|  |  | Easy if you are alone a lot and have your smartphone with you |
|  |  | Easy to follow EMA/EMI on weekends |
|  |  | Easier for students with no work routine who are at home |
|  | App use based on availability/possibility | “either did it or not" |
|  |  | The EMA/EMI timepoints have been adjusted so that they did not disturb |
|  |  | You can always trigger an EMA/EMI independently |
|  |  | Vs. often not triggered independently |
|  |  | When lack of time, just clicked through or skipped it |
|  |  | Request postponed |
|  | No usage in school/university/at work | Notification/Alarm disturbing |
|  |  | Smartphone is off during school or working hours |
|  |  | Therefore missed EMA/EMI |
|  | Not used in the wrong environment | Especially the breathing EMI |
|  | Difficult to conduct when “on the go” |  |
|  | Interest and usage adherence decreased over the course of the study | Made fewer EMA towards the end |
|  |  | Surprise effect of EMI was gone |
|  |  | “Automatism” when filling out the EMA |
|  |  | you already knew the questions by heart |
|  |  | the longer, the more inaccurate/superficial information |
|  |  | often answered the same thing |
|  |  | more free text or personalization could counteract |
| **3.2. Typical day with the app/AI4U training** | Favorite EMI was conducted more often | I followed my intuition |
|  | Fixed breaks were taken |  |
|  | AI4U training as regular companion |  |
|  | EMA used when “needed”/felt bad | Inappropriate when EMI came when you were feeling well |
|  |  | Automatism in filling out the EMA when things go well |
|  |  | EMA is also interesting in unfavorable situations |
|  |  | vs. No use if emotions were “really” bad |
|  |  | Here potentially more effective retrospectively than in an acute situation |
|  | EMA were rather conducted than EMI | EMI sometimes needed to much time to get involved with |
|  |  | EMA are easy/quick to fill out compared to EMI |
|  | EMA were time-consuming | User Interface could be improved - too many pages |
|  | Sometimes no EMI directly after an EMA | If an EMA is postponed by the user, then do not suggest an EMI |
|  |  | Integrating EMI directly into EMA would be better |
|  | Usage especially during peak times in the morning, noon, and evening | After school/before bed |
|  |  | EMI in the evening |
|  |  | vs. no more EMI in the evening |
|  |  | before schoolwork had to be completed |
|  |  | App not disturbing after school/at home |
|  |  | Before school/work |
|  |  | after the morning routine |
|  |  | start the day with the app as a routine |
|  |  | an EMA/EMI after waking up |
| **4. General evaluation of EMA** | The rationale for the content selection was not clear | Did not always capture emotional “peaks” |
|  |  | vs. selection was clear |
|  |  | Emotions difficult to scale (EMA) |
|  |  | Situations cannot always be classified |
|  |  | Activities difficult to define (EMA) |
|  | Wide range/selection of EMA | vs. it was "always the same" |
|  |  | Sometimes repetitive |
|  |  | Only difficult to fill out when it was an uneventful day |
|  |  | Enabled specific answers |
|  |  | vs. more variety/differentiation would be desirable |
|  |  | Potential answer options suited every mood |
|  |  | Amazing: Appropriate timepoints of EMA prompts (aligned with emotional states) |
|  | Missing content | Providing contextual information would be important for interpreting the dashboard later |
|  |  | EMA about anger |
|  |  | EMA about nervousness |
|  |  | EMA on motivation/boredom |
|  |  | EMA on physical well-being |
|  |  | EMA on extraordinary stress, such as exam stress |
|  |  | EMA on activity "I'm just on the move" |
| **5. General evaluation of EMI** |  |  |
| **5.1. Match of EMI to EMA/preferences** | Wide spectrum suitable for all life situations |  |
|  | Some EMI were proposed more often than others | the ones that I liked more/were more helpful |
|  | No temporal/personalized pattern was visible | I didn't think about it |
|  |  | not enough alignment with EMA answers |
|  |  | But that wasn't the expectation |
|  |  | vs. good fit between EMA answers and EMI prompts |
|  | Missing content | EMI for physical activity |
|  |  | I did not miss any other EMI |
|  |  | EMI for daily to-do planner |
| **5.2. Evaluation of EMI** | Not enough variation / surprise | vs. enough variety was given |
|  |  | avoided automatism |
|  |  | Unknown/new EMI were the most interesting |
|  | Breathing EMI: First have to “find your way around” | Vs. I practiced Breathing Exercises beforehand and was therefore easy to “get along with” |
|  | The Emotional Compass Intervention was difficult to follow | Meaning/effect was not apparent |
|  |  | not helpful |
|  |  | too many questions |
|  |  | overlapping with EMA |
|  | You had to “to drag something out” | This may have resulted in incorrect answers |
|  | Imagination interventions (EMI) are difficult and require imagination skills | I do not have the right mindset for “My Quite and Safe Place”: realistic type |
|  |  | Imagination improved over time |
|  |  | Imagination of “Emotions as a Wave” difficult |
|  |  | Difficult to think of a specific situation that would fit this imagination |
|  |  | vs. I physically "swayed along" |
| **6. Dashboard usage** |  |  |
| **6.1 Usage frequency** | Only during consultation appointments [hybrid]^[[3]](#footnote-3)^ | to avoid overthinking [hybrid] |
|  | Not that often | forgot to look it up |
|  |  | Reminder function would be helpful |
|  |  | Complicated because it is not integrated into the app |
|  |  | Less pressure/room for false statements/self-lies |
|  |  | Due to technical difficulties |
|  |  | Only useful when a trend was recognizable, with time intervals |
|  |  | helpful for “tracking” |
|  |  | don't need information about feelings of the last few days |
|  |  | only used towards the end |
| **6.2 Add-on value and benefit of the dashboard** |  |  |
| **6.2.1 Positive** | Emotions and situations (examples of learned health knowledge) | My reason for a negative mood is often school, exams, lessons |
|  |  | Trend visible in stressful phases |
|  |  | "Logically, when you write an exam, you are stressed" |
|  |  | I am more stable than I expected |
|  |  | I have very volatile emotions |
|  |  | My emotions are the result of the interaction of several factors |
|  |  | I am happier when with friends/out |
|  |  | I noticed that I wasn't getting enough sleep |
|  |  | Music cheers up my mood |
|  |  | Good sleep has a positive effect on other things and vice versa |
|  |  | Social conflicts reduce my self-esteem |
|  |  | I am more often alone than I expected |
|  | Professional support helpful [hybrid] | Helpful to reconsider emotions/life events |
|  |  | Vs. dashboard improved self-confidence since it was objective/neutral feedback |
|  | It's nice to have “Moments of Joy” listed in the dashboard |  |
|  | Helps to “suppress” emotions less |  |
|  | Helpful because you had a visible “result” |  |
|  | Search for causes or contextual factors of EMA via dashboard is “funny”/interesting |  |
|  | Helped to discover positive things (otherwise often focusing on negative things) |  |
|  | App would be boring/less approachable without dashboard |  |
|  | Helpful for awareness and reflection | Made you think about things |
|  |  | Dashboard like a personal diary |
|  | Development over time was “exciting” |  |
|  | Dashboard data was very consistent with emotions |  |
|  | Dashboard was interesting |  |
|  | Visualization in the dashboard was helpful |  |
| **6.2.2 Negative** | The more data in the dashboard, the more beneficial | “Just” EMA results are useless without context |
|  | Independent evaluation/interpretation was difficult | Amount of data overwhelming |
|  |  | vs. self-explanatory and intuitive |
|  |  | No connection between different EMA categories could be established |
|  |  | Evaluating dashboard data too time-consuming |
|  |  | Automatic interpretation would be helpful |
|  | No 'way out' suggested, when mood was negative | “What conclusions should I draw from this?” |
|  | Interesting, but did not yield in any changes | Connections that I already knew beforehand |
|  |  | I can assess my mood quite well (do not need dashboard for that) |
|  |  | I was looking more for the causes of my emotions |
|  |  | Did not explicitly look for connections |
|  |  | No added value, just interesting |
| **7. Subjective effect of the app** |  |  |
| **7.1. Improved regulation of emotions** | Deal with emotions better |  |
|  | Effect was mainly induced by the EMI |  |
|  | Calmed down (negative) emotions |  |
|  | Learned, that emotions are transient |  |
| **7.2. App „allowed“ to calm down** | Learned, that I need more time to wind down than I previously allowed myself | I am now sure that I can achieve everything |
|  | Helped to take a break and do something good for myself |  |
|  | Without the app this probably wouldn't have happened | App use was seen as a “task” (positive, since one had something “to do”) |
| **7.3. Calming down / stress reduction through EMI** | I have become more introspective |  |
|  | More relaxed/calm start into the day |  |
|  | Reduction of stress level |  |
|  | via „My Empathetic Companion” |  |
|  | via “My Quite and Safe Place” |  |
|  | via the „Breathing intervention“ | is calming but still "active" |
|  |  | helps to concentrate again |
| **7.4. Improved self-reflection** | Via the EMA | Better understand the reason for emotions |
|  |  | Classify/express emotions better |
|  |  | Increased awareness |
|  |  | Helped to recognize my emotions first |
|  |  | Helped to be more aware of the moment |
|  | Via the „Positive Data Log” |  |
| **7.5. Stimulates positive mindset** | „Moments of Joy“ and „Positive Data Log“ stimulated positive mindset | Things that are otherwise often overlooked in stressful everyday life |
|  |  | Noticed, that I can influence my happiness myself |
|  |  | Happiness about the little things in everyday life |
|  | Helped to “detach” from focus on negative things | vs. depressing when you can only enter negative things |
|  | positive memories through „My Quiet and Safe Place” |  |
|  | positive emotions through „My Empathetic Companion” | Triggered feeling of security |
| **7.6. Learned health knowledge /health literacy** | Sleep has an impact on many things |  |
|  | My emotions fluctuate a lot |  |
|  | Emotions always depend on the situation |  |
|  | I became aware that I have a lack of sleep |  |
|  | I became aware that I sometimes do not feel good (“mentally”) |  |
|  | I am more stress-resistant than expected |  |
|  | I feel less stressed/feel better during my leisure time |  |
|  | I have learned some coping strategies |  |
|  | I have learned new health-related theories |  |
| **7.7. Change in communication behavior**  *Response to the question: “Who do you talk to about feelings, regardless of the app?”* | Changes in communicating about emotions triggered through the app | I make fewer concessions with myself |
|  |  | EMA/EMI set impulses for conversations |
|  |  | I do not know |
|  |  | App induced no change in my communication behavior |
|  |  | Become aware of what you don't normally talk about |
| **7.7 Negative (side-)effects** | Through reflection, negative feelings become stronger |  |
|  | Perceived compulsion of app usage | I had to push myself sometimes |
|  |  | I felt “forced” to conduct an EMI |
|  |  | I felt a bit “pushed” by EMA |
|  |  | “felt bad” when rejecting EMA/EMI |
|  |  | EMA increased “pressure” to continuous fill them out thoroughly |
|  | [partially negative] App has “forced” you to reflect | But without the app this would not have happened |
|  | “Moments of Joy” depressing, if nothing came to my mind | I did not conduct the “Moments of Joy” intervention when I was sad |
|  | Notification/Alarm annoying | I developed a resistance |
|  |  | Mute function did not work |
|  | App usage has been stressful | Number of EMA was stressful |
|  |  | Using the app was overwhelming |
|  |  | vs. surprised that app usage was not stressful |
| **8. Trust in the app / limits** |  |  |
| **8.1 Trust in AI because not a “real” person** | Is not a stranger/ "human" | vs. difficult because app like “unfamiliar person” |
|  | Fear that friends/humans could misuse information |  |
|  | Lower inhibition threshold/easier to communicate |  |
|  | Allows for anonymity |  |
|  | "neutral diary" |  |
|  | Smartphones are already “everyday companions” |  |
|  | The smartphone is always available |  |
|  | Helpful for introverted people |  |
|  | If you have any problems, contact the app first rather than a professional | But: I do not want professionals to monitor my EMA entries |
| **8.2 Trust in AI4U training regarding data protection** | “only” AI / can’t “do anything” with the data anyway |  |
|  | Because developed by professionals and part of a research study |  |
|  | It doesn't matter, because there's already a lot of data on the internet anyway |  |
|  | Trust in data protection when no data is sold |  |
|  | No app use yet due to fear of data |  |
| **8.3 Consulting context** | - | - |
| **8.4 Else (Limits)** | Does not replace a hug from a friend |  |
|  | Does not replace helpful conversations with friends |  |
|  | Does not replace therapy |  |
|  | App is not a “miracle” |  |
|  | I can't imagine that AI adapts perfectly |  |
|  | Change: Smartphone that asks how you are doing (not annoying) |  |
|  | Trust that the app can help you |  |
|  | Users have to have a willingness to reflect/work on themselves |  |
|  | App may not “prescribe” (control/limit) | I would drop out of the study if the app would have forced towards self-optimization |
|  |  | I decide for myself, how much I share |
| **9. Permanent effects of use** |  |  |
| **9.1. Sustained effects** |  |  |
| **9.1.1. More reflection in everyday life** | I reflect more in everyday life |  |
|  | I think more about the positive/important things |  |
|  | It made me more goal-oriented |  |
| **9.1.2. Relaxation / calming down** | I calm down using EMI |  |
|  | I continue to do the breathing EMI | helpful for winding down during stressful times |
|  | I have more stable mood and I am calmer in everyday life | I take more breaks |
|  |  | I start the day in “peace” |
| **9.1.3. Understand emotions better** | Emotions are transient (especially through emotions as a wave) |  |
|  | Sharing feelings is now easier |  |
|  | mHealth app as an opportunity to talk more openly about feelings | especially if several friends use the app - exchange! |
|  |  | But: my friends are not open to mHealth |
|  |  | mHealth is no longer a “taboo topic” for the younger generation |
| **9.2. Reasons for little change in habits** | Evaluation of the long-term effect is pending (only recently completed the study period) |  |
|  | No change in “lifestyle” possible | Can only be achieved in the long term when better adaptable to everyday life |
|  |  | more control/self-determination |
|  |  | Fewer EMA/EMI prompts |
|  |  | Depending on the situation, difficult to apply/implement in everyday life |
|  |  | "especially at my age" |
|  |  | rather when you have enough leisure time |
|  |  | becomes more difficult with increased stress |
|  | Habits have not yet become firmly established | Study period too short |
|  |  | “mentally” completed after the end of the study |
|  |  | Without an app it is more likely to forget new routines/ conduct at lower intensity |
|  |  | you would need a solid plan to remember |
|  |  | you would have to be disciplined enough, to keep doing it |
|  |  | you don't take enough time for yourself |
| **9.3 Ways of sustaining learned coping strategies** | Exercises/reflection could also be integrated without an app | vs. I miss the EMA |
|  |  | I conduct my favorite EMI also without the app |
|  |  | but at a lower frequency |
|  |  | “Analogue” media is preferred over digital input |
|  |  | reduces distraction |
|  |  | reduces media consumption |
| **10. Open discussion points** |  |  |
| **10.1 Technical aspects** | You can't jump back during EMI |  |
|  | Docking headphones for audio EMI |  |
|  | Technical error, EMA prompt at midnight |  |
| **10.2 Design questions/comments** | No effect suspected due to “study smartphone” | Using your own smartphone would improve daily life integration |
|  | Intuitive user interface |  |
|  | Rudimentary design |  |
|  | Gamification elements are motivating |  |
|  | AI could create an user profile/peer group and base EMI suggestions on that |  |
|  | Visualize your own feelings in EMI/EMA |  |
|  | It would be good to ask about the location so that the app can select the appropriate EMI |  |
| **10.3 Else** | “Moments of Joy”/”Success Diary” would especially be helpful before falling asleep |  |
|  | “Dehumanization” helpful for “male” people? |  |
|  | Would recommend the app to friends who are more melancholic people |  |
|  | Great message from the app: “I want to help you” |  |

Coding tree of focus groups^[[4]](#footnote-4),^^[[5]](#footnote-5)^

| **Thematic (sub-) categories** | **Codes** | **Subcodes** |
| --- | --- | --- |
| 1. **Socio-demographic data** |  |  |
| 1. **Integration of the app into everyday life** |  |  |
| - 1. **Handling of EMA and EMI** | Difficult to implement/uncomfortable in front of friends/other people |  |
|  | Preference to have EMA/EMI prompts when feeling bad |  |
|  | EMA are better to integrate into daily life than EMI |  |
|  | Dependent on timing/daily rhythm | Difficult at work |
|  |  | Easier during flexible everyday life (i.e., home office) |
|  |  | doesn't fit into a full daily schedule |
|  |  | Vs. easier with a regulated routine |
|  |  | Usage is difficult when on the go |
|  |  | I simply 'clicked through' and collected points |
|  |  | Challenging during school/university |
|  |  | Because I am surrounded by people/not alone |
| - 1. **Suggestions for improved daily life integration** |  |  |
| - - 1. **Control: Shared decision-making & Self-selection** | [participatory] option to indicate personalized availability / time slots | [participatory] more flexible option to "postpone" would be helpful |
|  |  | Fixed pre-set timepoints would enhance integration |
| - - 1. **[participatory]^[[6]](#footnote-6)^ time-consuming app usage** | Dashboard too time-consuming |  |
|  | Too many EMA per day | [participatory] unrealistic to answer everything |
|  |  | "10-day introductory period" is burdensome |
|  | Second study phone is burdensome | Notification/alarm is disturbing |
|  |  | I forgot the study smartphone |
|  |  | Burdensome to carry the second phone |
|  | [participatory] EMI are too lengthy and partly repetitive | [participatory] shorter EMI would be easier to integrate |
|  |  | [participatory] Offering shortened versions (i.e. two versions) of both EMI and EMA would be beneficial |
|  |  | Some level of detail in the EMA is unnecessary |
|  |  | [participatory] difficulties in maintaining focus |
|  |  | vs. [participatory] it trained my patience |
| 1. **Subjective effect of EMA/EMI** |  |  |
| - 1. **Pros/Cons of the EMI** |  |  |
| - - 1. **“Moments of Joy” and “Positive Data Log”** | [participatory] positive to actively self-reflect and provide “input” |  |
|  | Easy to understand |  |
|  | Easily integrated into daily life |  |
|  | “Positive Data Log” resembles "productivity measurement" (negative impact) |  |
|  | “Moments of Joy” pleasant to reflect | particularly helpful towards the end of the day |
|  | Stimulates positive affect | beneficial to reflect on snapshots during the day |
|  |  | helpful to reflect on the minor good things |
| - - 1. **Emotions as Wave** | Emotions are transient |  |
|  | “Emotion as a Wave” was not “felt” | wave analogy is a misrepresentation |
| - - 1. **Breathing Intervention** | Breathing intervention helped to calm down |  |
|  | [participatory] "Training effect“, the more frequent, the more helpful |  |
|  | Something more „physical“ |  |
|  | Easily integrated into daily life | however, not conducted after study period |
|  |  | Vs. implemented as a coping strategy |
| - - 1. **Imagination-based interventions** *(My Empathetic Companion and My Quit and Safe Place)* | [participatory] difficult to imagine and time-consuming | requires training / getting used to at first |
|  |  | Vs. good to have a visual mental stimulus |
|  |  | however, requires one's own imagination |
|  |  | vs. Imagination-based interventions simple and nice variety |
|  | Independent of location and able to conduct anywhere | vs. requires a certain environment |
|  |  | vs. not applicable in front of other people |
|  |  | [participatory] limited benefit when at home (“it is already my safe place”) |
| - - 1. **Emotional Compass** | Was confusing |  |
|  | An application example would have been helpful |  |
|  | Difficult to apply /transfer to one´s own situation |  |
|  | Too abstract | vs. emotion analysis is interesting |
| - - 1. **Other** | EMI help to distance oneself from challenging situations |  |
|  | Difficult to apply, "so what?!" |  |
|  | App-usage is burdensome / exhausting | App-usage as stress factor in everyday life |
|  | App is prescriptive | Fear of missing out on EMA |
|  |  | At times, one felt compelled to participate |
| - 1. **Reflection /awareness through EMA** | Reduces the need of other forms of exchange |  |
|  | Would not have happened otherwise in daily life |  |
|  | Understanding the reason behind emotions |  |
|  | Regular reflection |  |
|  | Focus on the good things |  |
|  | Difficult to quantify emotions as a numerical value |  |
|  | Increased emotional awareness |  |
|  | Reflecting has become a sustainable part of daily life |  |
| - 1. **Missing content** | No, because the EMA/EMI offers were enough |  |
|  | Explanation of the EMI / its effects |  |
|  | EMA on physical discomfort |  |
|  | EMI for physical activity | "Power pose" / short movement or stretching |
| 1. **Feedback on digital monitoring (dashboard)** |  |  |
| - 1. **Low dashboard usage frequency / Usage barriers** | Changes only noticeable after a period of time |  |
|  | Lack of user-friendliness, unclear layout | Only external on the laptop, integration into the app would be more useful |
|  |  | [Participatory] Numerous data points make it unclear |
|  |  | Non-intuitive controls/statistics |
|  |  | vs. very clear graphical representation |
| - 1. **Limited independent interpretation of dashboard data** | Learned health-related knowledge | I feel more positive when interacting with people |
|  |  | Fatigue and mood interplay |
|  | [participatory] provides only a superficial / rough overview | Tabels were not interesting |
|  |  | Relationships / connections between EMA were not recognized |
|  |  | Too little time for interpretation |
|  |  | Needs to be simpler / more accessible |
|  |  | Professional guidance might be helpful |
|  | [participatory] A summary would be desirable | Getting interpretation rather than searching for it oneself |
|  |  | Daily summary function |
| 1. **Intervention selection** |  |  |
| - 1. **AI-based assignment of interventions** | AI was not noticed | Didn't accurately recognize when one was feeling bad/good |
|  |  | More a kind of random selection |
|  |  | AI didn't recognize individual preferences |
|  | AI is „irrelevant“ / does not matter |  |
|  | Became more precise over time |  |
| - 1. **Reasons for lack of EMI suitability** | Daily life is too irregular |  |
|  | Too little variety |  |
|  | Not the right time / place | “Moments of Joy”/”Positive Data Log” in the morning are inappropriate |
|  |  | Suggested EMI in the wrong moment |
| 1. **[Hybrid]^[[7]](#footnote-7)^ Feedback on follow-up discussions / consultation appointments** |  |  |
| - 1. **Not yet discussed with counselor** |  |  |
| 1. **Evaluation and reflection on AI-informed mHealth apps** |  |  |
| - 1. **Attitude towards data security** | Not as "invasive" (e.g. like other algorithms on social media) |  |
|  | I want to control my daily data disclosure |  |
|  | The benefits don't justify data disclosure |  |
|  | Only acceptable within the scope of a study |  |
|  | As long as data is processed securely /transparently |  |
| - 1. **AI decision-making processes and control** | Demand for shared decision-making & self-selection of content | Feedback option for the EMI |
|  |  | Uncertain if AI is perfectly capable of capturing emotions |
|  | Relying on the decision-making ability of AI | I do not expect it to function perfectly |
|  |  | I will not be able to teach the AI much more |
|  |  | Vs. I feel overwhelmed with too much self-determination (trade-off) |
|  |  | "Only" feeding AI with data requires little effort |
| - 1. **Opportunities and barriers for a trustworthy AI** | Lower threshold to disclose emotions |  |
|  | I do not have to look anyone in the eye [Trust] |  |
|  | It takes time getting used to it, but fades once the benefit gets visible |  |
|  | Unusual at first, to disclose emotions in an app |  |
| - 1. **Long-term effects and implementation conditions** | No sustainable improvement in well-being yet |  |
|  | Stress may be lower if not part of a study |  |
|  | AI and personalization should be better | Desire for more variety (expectation) |
|  |  | "Expected more" from EMI (expectation) |
| 1. **Open discussion points** | Intuitive user interface |  |
|  | [participatory] Audio description of EMI could be more relaxing than reading them |  |
|  | Design needs improvement | Improve gamification elements |
|  | Technical aspects | [participatory] Technical issue: Limited mute function |
|  |  | hence not used much while on the go |
|  |  | Technical: EMA were not saved in the dashboard during the first week (was solved after consultation with study personnel) |
|  | Potential dialogue population for a mHealth app | Openness and willingness to reflect are necessary |
|  |  | For individuals without a trusted person/ introverted individual |
|  |  | Media affinity is necessary |

1. The data analysis was conducted in German and coding trees later translated into English [↑](#footnote-ref-1)
2. The coding tree depicted contains all categories from our data analysis. Thematic categories derived from the interview topic guide and emerged subcategories from the transcripts are shown in level 1 and 2. Level 3 includes emerged codes for each of the subcategories and Level 4 and 5 shows respective subcodes. [↑](#footnote-ref-2)
3. „[hybrid]“ indicates that this category is only coded, if it was a participant having optional sessions with counsellors or receiving care from educational counselling services and he/she said something specific to e.g. the consultation appointments. [↑](#footnote-ref-3)
4. The data analysis was conducted in German and coding trees later translated into english [↑](#footnote-ref-4)
5. The coding tree depicted containts all categories from our data analysis. Thematic categories derived from the interview topic guide and emerged subcategories from the transcripts are shown in level 1 and 2. Level 3 includes emerged codes for each of the subcategories and Level 4 and 5 shows respective subcodes. [↑](#footnote-ref-5)
6. “[participatory]” indicates that this thematic (sub-)categorie or (sub-)code emerged via our participatory research workshop with our “Co-Researchers” [↑](#footnote-ref-6)
7. „[hybrid]“ indicates that this category is only coded, if it was a participant having optional sessions with counsellors or receiving care from educational counselling services and he/she said something specific to e.g. the consultation appointments. [↑](#footnote-ref-7)
